# Altered cortical synaptic lipid signaling leads to intermediate phenotypes of mental disorders

**DOI:** 10.1038/s41380-024-02598-2

**Published:** 2024-05-28

**Authors:** Oliver Tüscher, Muthuraman Muthuraman, Johann-Philipp Horstmann, Guilherme Horta, Konstantin Radyushkin, Jan Baumgart, Torfi Sigurdsson, Heiko Endle, Haichao Ji, Prisca Kuhnhäuser, Jan Götz, Lara-Jane Kepser, Martin Lotze, Hans J. Grabe, Henry Völzke, Elisabeth J. Leehr, Susanne Meinert, Nils Opel, Sebastian Richers, Albrecht Stroh, Silvia Daun, Marc Tittgemeyer, Timo Uphaus, Falk Steffen, Frauke Zipp, Joachim Groß, Sergiu Groppa, Udo Dannlowski, Robert Nitsch, Johannes Vogt

**Affiliations:** 1 Department of Psychiatry and Psychotherapy, University Medical Center of the Johannes Gutenberg-University Mainz, Mainz, Germany; 2 Department of Neurology, Johannes Gutenberg-University Mainz, Mainz, Germany; 3 Department of Neurology, Neural engineering with Signal Analytics and Artificial Intelligence (NESA-AI), University Hospital of Würzburg, Würzburg, Germany; 4 Informatics for Medical Technology, University Augsburg, Augsburg, Germany; 5 Focus Program Translational Neuroscience, Johannes Gutenberg-University Mainz, Mainz, Germany; 6 TARC, Translational Animal Research Center, University Medical Center of the Johannes Gutenberg University Mainz, Mainz, Germany; 7 Institute of Neurophysiology, University Medical Center, Goethe-University Frankfurt, Frankfurt, Germany; 8 Department of Molecular and Translational Neuroscience, Institute of Anatomy II, Cluster of Excellence-Cellular Stress Response in Aging-Associated Diseases (CECAD), Center of Molecular Medicine Cologne (CMMC), University of Cologne, Cologne, Germany; 9 Functional Imaging Unit, Diagnostic Radiology and Neuroradiology, University Medicine Greifswald, Greifswald, Germany; 10 Department of Psychiatry and Psychotherapy, University Medicine Greifswald, Greifswald, Germany; 11 Department SHIP/Clinical Epidemiological Research, Institute of Community Medicine, University Medicine Greifswald, Greifswald, Germany; 12 Institute for Translational Psychiatry, University of Münster, Münster, Germany; 13 Institute for Translational Neuroscience, University of Münster, Münster, Germany; 14 Institute of Pathophysiology, Johannes Gutenberg-University Mainz, Mainz, Germany; 15 Cognitive Neuroscience, Institute of Neuroscience and Medicine (IMN-3), Research Centre Jülich, Jülich, Germany; 16 Max Planck Institute of Metabolism Research, Cologne, Cluster of Excellence-Cellular Stress Response in Aging-Associated Diseases (CECAD), Cologne, Germany; 17 Institute for Biomagnetism and Biosignalanalysis, University of Münster, Münster, Germany; 18 Present Address: Leibniz Institute for Resilience Research Mainz, Mainz, Germany; 19 Present Address: Institute for Molecular Biology Mainz, Mainz, Germany; 20 Present Address: Institute of Anatomy, University Medical Center Mainz, Mainz, Germany

**Keywords:** Psychiatric disorders, Neuroscience, Predictive markers

## Abstract

Excitation/inhibition (E/I) balance plays important roles in mental disorders. Bioactive phospholipids like lysophosphatidic acid (LPA) are synthesized by the enzyme autotaxin (ATX) at cortical synapses and modulate glutamatergic transmission, and eventually alter E/I balance of cortical networks. Here, we analyzed functional consequences of altered E/I balance in 25 human subjects induced by genetic disruption of the synaptic lipid signaling modifier PRG-1, which were compared to 25 age and sex matched control subjects. Furthermore, we tested therapeutic options targeting ATX in a related mouse line. Using EEG combined with TMS in an instructed fear paradigm, neuropsychological analysis and an fMRI based episodic memory task, we found intermediate phenotypes of mental disorders in human carriers of a loss-of-function single nucleotide polymorphism of *PRG-1* (*PRG-1*^*R345T/WT*^*)*. *Prg-1*^*R346T/WT*^ animals phenocopied human carriers showing increased anxiety, a depressive phenotype and lower stress resilience. Network analysis revealed that coherence and phase-amplitude coupling were altered by *PRG-1* deficiency in memory related circuits in humans and mice alike. Brain oscillation phenotypes were restored by inhibtion of ATX in *Prg-1* deficient mice indicating an interventional potential for mental disorders.

## Introduction

Alterations in glutamatergic transmission influencing cortical network excitability and changing excitation/inhibition (E/I) balance have been shown to be of critical importance for psychiatric disorders [1–3]. Recent data implicates synaptic bioactive phospholipids, such as lysophosphatidic acid (LPA) in homeostatic regulation of glutamatergic transmission [4, 5], modulation of cortical network excitability and E/I-balance, all of which were involved in mental disorders [6], feeding behavior [7] and stroke pathophysiology [8]. LPA is a short-lived but potent signaling molecule acting via specific G-protein coupled receptors (LPA-receptors 1–6 [9]) and is locally synthesized by autotaxin (ATX) at the synaptic cleft, where it activates high-affinity LPA_2_-receptors at presynaptic terminals [6]. Activation of LPA_2_ receptors increase glutamatergic release probabilities and thereby excitability of postsynaptic neurons changing cortical network excitability and E/I balance [4]. *Plasticity-related gene 1* (*PRG-1/PLPPR4*) codes for an LPA-interacting synaptic protein (PRG-1/LPPR4), which belongs to the superfamily of lipid-phosphate phosphatases (LPPs). PRG-1 is found in spines of cortical excitatory neurons and is located at the postsynaptic density of excitatory synapses. Via its ability to internalize LPA into cells and thereby to remove it from the synaptic cleft [4, 10], PRG-1 controls the effect of LPA-stimulation and thus the activity of presynaptic LPA_2_-receptors [4]. By controlling synaptic LPA-levels, PRG-1 regulates glutamate transmission and excitability of cortical neurons and their neuronal networks (see Fig. S1).

In a retrospective data analysis, we have previously identified human individuals, which carried a single nucleotide polymorphism (SNP) in one allele of the *PRG-1* gene (rs138327459, *PRG-1*^*R345T/WT*^) and displayed an altered cortical auditory information processing. *PRG-1*^*R345T*^ SNP is a loss-of-function mutation impairing LPA-uptake from the synaptic cleft and affecting sensorimotor gating [10]. Genetically modified mice expressing a homolog of this human mutation in the *Prg-1* gene (*Prg-1*^*R346T*^) showed signs of cortical hyperexcitability and altered pre-pulse inhibition, a mouse correlate of sensorimotor gating [6]. These data suggest that altered LPA signaling at cortical synapses might have significant implications for proper function of the human cortex. Since translational evidence from synaptic level to cortical networks and from mice to man is scarce, we conducted a prospective study in a cohort of individuals carrying the *PRG-1*^*R345T/WT*^ mutation and compared them to *Prg-1* deficient animal models. We first analyzed effects of altered synaptic lipid signaling in humans on cortical network excitability and changes in E/I balance, which are supposed to play critical roles in psychiatric disorders. Specifically, we analyzed brain oscillations by high-density EEG together with extracranial stimulation (transcranial magnetic stimulation, TMS) in combination with a conditioned fear paradigm and analyzed changes in cortical network activity in *PRG-1*^*R345T*^ expressing human subjects and sex and age matched control subjects. Finally, we analyzed phenotypical consequences of shifted E/I balance in men and in an animal model with regard to memory formation, behavior, and possible therapeutic interventions.

## Methods

### Human studies

#### Ethics

This study was conducted at the university hospitals of Mainz, Germany, and Greifswald, Germany, and was approved by the Institutional Review Board of the University of Greifswald and by the local Ethics committee of the State Medical Association Rhineland-Palatinate Mainz, Germany, and complies with the declaration of Helsinki [11]. Healthy human volunteers were recruited from population-based cohort studies (Study of Health in Pomerania: SHIP and SHIP-Trend in Greifswald, and the Gutenberg Brain Study [GBS] in Mainz, Germany) as previously described [12–14]. Genotyping for wildtype or *PRG-1*^*R345T/WT*^ carrier status was determined by gene sequencing in the GBS cohort or by whole-genome sequencing in the SHIP and SHIP-Trend cohorts. Controls were selected as individuals each matching to a respective Gene Carrier in sex, education, and ±10 years of age. Inclusion criteria were 18–80 years of age, fluency in German, normal or corrected eyesight. Exclusion criteria were cognitive disability, lifetime diagnosis of substance dependence, lifetime diagnosis of schizophrenia, lifetime diagnosis of bipolar disorder, lifetime diagnosis of organic mental disorder, current psychiatric disease, substance abuse (except nicotine) or psychiatric medication during the last six months as well as major neurologic conditions such as Parkinson’s disease, Multiple Sclerosis or past stroke. Informed consent for the study and scientific use of the data was acquired in written form from each participant before inclusion (for additional details see Supplementary Methods). From the above mentioned cohorts, 25 human *PRG-1*^*R345T/WT*^ mutation carriers (rs138327459) and 25 age and sex-matched control subjects were analyzed in this study (see also Table 1 and Supplementary Table S1 for more details).

**Table 1:** Sample characteristics and neuropsychiatric measures.

		control subjects	*PRG-1*^*R345T/WT*^	significance	group differences	effect size (mode)
subjects		25	25			
Site (Greifsw./Mainz)		19/6	19/6			
Sex (Male/Female)		8/17	8/17			
Handness (right/left)*		19/-	22/2			
Age (years)		52.8 ± 13.6	53,3 ± 12,8	n.s.	53.1%	0.05
Education (years)		10,7 ± 1,2	10,6 ± 1,1	n.s.	55.3%	0.03
IQ (MWT-B)		110,4 ± 8,7	111,9 ± 8.7	n.s.	74.1%	0.20
WAIS-MR		17,8 ± 6,1	18,0 ± 4,3	n.s.	51.8%	0.02
WAIS-BD		45,6 ± 12,2	43,8 ± 10,2	n.s.	71.9%	0,18
PASAT (z-scores)		0.15 ± 1.0	−0.16 ± 1.1	significant	81.1%	0.25
TAP alertness (reaction time in ms, RT) (reaaction time variation, RTV)	Tonic Alertness-RT	273,7 ± 35,1	266,1 ± 32,3	n.s.	78.3%	0.22
	Phasic Alertness-RT	268,1 ± 33,4	262,1 ± 32,4	n.s.	76.1%	0.19
	Tonic Alertness-RTV	34,5 ± 10,8	34,1 ± 11,3	n.s.	57.4%	0.06
	Phasic Alertness-RTV	34,2 ± 12,0	36,3 ± 12,2	n.s.	71.4%	0.18
	RT-Diff. phasic & tonic alertness	5,6 ± 24,0	4,0 ± 12,3	n.s.	56.8%	0.05
TAP flexibility (reaction time in ms, RT) (reaaction time variation, RTV)	RT changing hands	758,4 ± 223,7	689,0 ± 160,5	n.s.	73.8%	0.19
	RT not changing hands	862,7 ± 276,8	744,8 ± 189,8	highly significant	94.8%	0.50
	overall RT	838,5 ± 263,6	733,6 ± 174,6	highly significant	93.8%	0.48
	RTV changing hands	180.8 ± 96,6	157,7 ± 69,6	n.s.	75.6%	0.21
	RTV not changing hands	227,8 ± 99,9	177,4 ± 69,9	highly significant	96.5%	0.54
	RT changing/no changing hands	0.89 ± 0,08	0.94 ± 0.09	highly significant	94.2%	0.50
	RT changing hands/overall	0.91 ± 0.05	0.94 ± 0.06	highly significant	94.9%	0.50
	Correct rate changing hands	29,8 ± 1.4	30,7 ± 0,6	highly significant	99.8%	1.17
	Correct rate no changing hands	64,3 ± 5,6	66,5 ± 2,2	highly significant	96.4%	0.62
	Index of overall performance	4,6 ± 10,2	9,9 ± 7,4	highly significant	96.2%	0.53
TAP shared attention (reaction time in ms, RT) (reaaction time variation, RTV)	Auditory RT	542.4 ± 73.7	518.6 ± 81.6	significant	81.9%	0.28
	Auditory RTV	146.4 ± 70.7	122.6 ± 48.3	highly significant	90.0%	0.44
	Auditory correct rate	18.9 ± 1.4	19.3 ± 1.3	significant	84.6%	0.34
	Visual RT	516.2 ± 66.1	516.1 ± 42.6	n.s.	54.2%	0.03
	Visual RTV	75.6 ± 24.7	77.1 ± 23.4	n.s.	62.9%	0.09
	Visual correct rate	20.0 ± 0,2	19.8 ± 0.8	n.s.	50.3%	0.01
	Index (Na/Ta)/(Nv/Tv)	0.95 ± 0.1	0.97 ± 0.0	highly significant	91.8%	0.45
Rey-Osterrieth Figure Test (ROF) (z-scores)	copy	−6.6e-16 ± 1	−2.0e-16 ± 0.9	n.s.	56.0%	0.04
	immediate recall	−0.04 ± 1.18	−0.064 ± 1.22	n.s.	50.8%	0.00
	delayed recall	−0.21 ± 1.24	−0.512 ± 1.40	significant	86,5%	0.31
Verbal Learning Memory Test (VLMT; z-scores)	VLMT early recall (1)	1,78 ± 1,20	1,14 ± 0,82	highly significant	97.4%	0.60
	VLMT late recall (5)	0,9 ± 0,8	0,6 ± 0,6	n.s.	51.1%	0.01
	VLMT sum (1–5)	1.35 ± 0,7	0.95 ± 0,8	highly significant	94.1%	0.47
	VLMT recognition	0,37 ± 0,6	0,44 ± 0,5	n.s.	56.2%	0.08
Episodic Memory Test (z-values)	recall, hits	−2.0e-07 ± 1,0	−4.348e-10 ± 1,0	n.s.	50.5%	0.006
	recognition, hits	−8.8e-07 ± 1,0	0 ± 1,0	n.s.	53.4%	0.069

Values represent means ± SD.

*data missing from 7 subjects.

### General neuropsychological assessment

*PRG-1*^*R345T/WT*^ carriers and age and sex-matched controls were subjected to a general neuropsychological assessment, which was performed before proceeding with the EEG/TMS threat processing test or with the fMRI episodic memory test. If not otherwise mentioned, 25 human *PRG-1*^*R345T/WT*^ mutation carriers and 25 age and sex-matched control subjects were included for neuropsychological assessment, which contained the German Mehrfachwahl Wortschatz Intelligence Test (MWT) [15], the German Wechsler Adult Intelligent Scale (WAIS) IV [16], the State-Trait Anxiety Inventory (STAI; 12 control subjects and 12 human *PRG-1*^*R345T/WT*^ mutation carriers) [17], Positive and Negative Affect Schedule (PANAS; 21 control subjects and 22 human *PRG-1*^*R345T/WT*^ mutation carriers) [18], the Rey-Osterrieth Figure Test (ROF) [19], and the verbal learning memory (VLMT) [20], a german version of the Rey Auditory Verbal learning Task. Attention performance (TAP) was assessed with different tests: TAP-alertness, TAP-flexibility and TAP-shared attention. Briefly, attention tasks were used from the computerized Test of Attentional Performance 2.3.1 (TAP) attention test battery [21] (for more details see Supplementary Methods). Results from neuropsychological analyses were corrected for age and sex.

### Analysis of global mean field power (GMFP) changes following TMS

The GMFP is an index of distinctive cortical excitability and also reflects the synchronous activity across observations in response to a specific stimulus [22, 23]. Following single, inhibitory double-TMS pulses (short-interval intracortical inhibition [SICI] with an interstimulus interval of 4 ms), CS- or CS+ presentation, GMFP was calculated for the time (−0.25 to 2 s) after TMS application. We assessed the local cortical excitability by estimating the GMFP specifically in the frontal cortex (16 frontal electrodes pooled together from the 10–10 channel EEG configuration).

### Instructed/conditioned fear task with concomitant dmPFC-TMS

The instructed fear paradigm was developed using the Cogent toolbox (http://www.vislab.ucl.ac.uk/cogent_2000.php) in Matlab R2006b (The MathWorks). Participants were instructed that the screen appearance of a circle (CS+) was associated with a probability of 33% of receiving an electro-dermal stimulation, while screen appearance of a square (CS-) was not associated with any threat. To evaluate the involvement of the dmPFC in threat processing and network dynamics, application of single TMS pulses over the right dmPFC was applied 1 s after stimulus (CS+ and CS- conditions) onset. The paradigm consisted of 100 stimuli (54 CS+, 46 CS-). For additional details see Supplementary Methods section.

### EEG-resting state microstate analysis

The topographies of the EEG signal were calculated at time points corresponding to global field power (GFP) peaks. These topographies were clustered into 4 prototype classes, which were back fitted to the full EEG data in PRG and HC to classify each segment of the data into one of 4 microstates. For each microstate class (A,B,C,D), length of individual instances namely duration, number of instances namely occurrence, and proportion of the full signal spent in the class namely coverage was calculated and compared between control subjects and *PRG-1*^*R345T/WT*^ mutation carriers using a Bayes one-way ANOVA (for group differences and effect size see also Supplementary Table 2).

### Power and coherence analyses

Coherence reveals components that are common to two signals in the frequency domain. Let two concurrently recorded signals be $x(t)$ and $y(t)$ of length N. The arithmetic mean is set to zero and the standard deviation is set to one for both data sets. The data sets of length N were divided into M disjoint segments of length L, so that N = LM. Power spectra ${S\_xx}$ (*ω*) and ${S\_yy}$ (*ω*) of the signals $x$ and $y$ are computed as the Fourier transform of the autocorrelation function in each window. The cross spectrum ${S\_xy}$ (*ω*) is the Fourier transform of the cross-correlation function of signal x and y in each window. Power spectra and cross spectrum are averaged across all segments to get an estimate of the same. The coherence measures the linear time invariant (LTI) relationship between two signals *x*(*t*) and *y*(*t*). The confidence limit which indicates the significance of the coherence at a particular frequency is calculated at the 100% $\alpha$ as given by 1-〖(1 − α) 〗${\wedge} \, (1 / ((M-1)))$ [24, 25] where $\alpha$ is set to 0.99, so the confidence limit is 〖1 − 0.01〗${\wedge} \, (1 / ((M-1)))$. We estimated the theta (4–8 Hz) and gamma power (30–100 Hz) in the entorhinal and the hippocampus from both men and mice.

### Cross-frequency coupling

The individual source signals from the entorhinal for the low frequency theta (4–8 Hz) and high frequency namely gamma (30–100 Hz) from the hippocampus were used for the estimation. First, a mix signal was estimated which is combination of the low-frequency signal, the high-frequency signal, and the auxiliary signal with an offset, respectively. The mix signal was processed with a bandpass, which was applied around the gamma frequency. This was followed by the Hilbert transform of the low frequency signal. At the end, the calculation of the amplitude envelope of the analytic signal was obtained. In this way, the phase to amplitude coupling was estimated between the two regions as previously described [26].

### fMRI-Episodic memory task (face-profession association task)

Episodic memory tasks were performed during fMRI scanning based on an established paradigm used in imaging genetics as described elsewhere [27, 28]. During the encoding task, participants were presented 16 face-profession pairs and 24 head contours as control condition. Participants had to imagine the presented person acting in the given profession. Following this, participants were asked to state if the profession suited the person’s face to induce deep encoding. In the recall phase, faces were presented again. Participants were asked, whether the depicted person had to complete apprenticeship or academic studies to execute the respective profession presented during the encoding stage (for further details see Supplementary Methods).

### Animal studies

*Husbandry*: all animal procedures were conducted in compliance with protocols approved by the local authorities (Landesuntersuchungsamt Rheinland-Pfalz). C57Bl/6J were obtained from Janvier, France. *Prg-1*^*R346T*^ and *Prg-1*^*−/−*^ male transgenic mice were generated as previously described and were genotyped accordingly [4, 6]. Animal numbers for each experiment sample size estimation was performed based on common practice in animal behavioral experiments. Sample sizes were chosen to generate reproducible experiments with a significance of *p* < 0.05 at a power of ≥0.8 and were calculated according to published literature [29]. Number of animals used in each experiment are mentioned in the figure legends and individual values are shown in the graphs.

In-vivo electrophysiological recordings were performed as previously described [30]. Electrophysiological recordings in awake, behaving animals were acquired using a 16-channel head stage and a Digital Lynx data acquisition system (Neuralynx, USA). To extract local field potentials, the neural signals were bandpass filtered between 1 and 1000 Hz and digitized at 2 kHz. Recording location was verified after animal perfusion in cresyl violet stained sagittal cut brain slices. In this experiment 13 *Prg-1*^*−/−*^ mice, 7 *Prg-1*^*−/−*^ mice + ATX-inhibition, and 15 WT litters were used.

### Power and coherence field potential analysis

Analyses of neuronal activity coherence between the MEC and in the CA1 was computed using the multitaper method (MATLAB routines provided by K. Harris, University College London, London, UK, and G. Buzsáki, New York University, New York, NY, USA). For oscillatory power analysis, the local field potential (LFP) signals were convolved with a series of Morlet wavelets with center frequencies ranging from 1 to 100 Hz and a length of three cycles. Power was than calculated by taking the absolute value of the wavelet transform and averaging across time to obtain the power spectrum. For statistical analysis, coherence and power were averaged within the theta (7–12 Hz) and gamma (30–100 Hz) frequency ranges. Underlying data for low gamma oscillations (30–70 Hz) was used for coherence analysis and partially published previously [6].

### Cross-frequency coupling

Calculation was performed on how the power of the LFP signal from CA1 at frequency *y* varied as a function of the phase of the signal from MEC at frequency *x*. The magnitude of cross-frequency coupling between MEC at frequency *x* and CA1 power at frequency *y* was defined as [max(*p*)-min(*p*)]/max(*p*). To examine coupling between MEC theta and CA1 gamma specifically, we used the above procedure to calculate the modulation of average 30-100 Hz power in CA1 by 10 Hz phase in MEC. The same procedure was used to examine coupling in the other direction, between CA1 theta and MEC gamma.

### Pharmacological treatment

Animals were treated using the in-vivo potent autotaxin inhibitor PF 8380 (Cayman Chemical Company, Ann Arbor, MI, USA) at a dose of 30 mg/KG body weight for treatment as described [6].

### Behavioral analyses

Behavioral analyses in the open field, fear conditioning, social interaction, sucrose preference, tail suspension, and social interaction after chronic social defeat stress were performed according to standard procedures (for further details see Supplementary Methods). Animals were tested in a random order to avoid systematic bias. Investigators were blinded for animal genotype, which was disclosed at the end of the experiment.

### Statistics using Bayesian analyses

For data with a non-Gaussian distribution and unequal sample sizes, we used Bayesian posterior distribution analyses to identify differences between the two groups. This analysis provides complete distributions of credible values for group means and their differences [31]. We have used the Markov Chain Monte Carlo (MCMC) approach to compute the Bayes factor for the choice of priors and the default MCMC sample size of 100,000. Differences of the means above 80% as well as an effect size above 80% were considered significant, differences of the means above 90% as well as an effect size above 90% were considered highly significant [32]. The mode of the effect size was calculated using following formula: (µ_1_-µ_2_)/√(σ_1_^2^ + σ_2_^2^)/2. This type of analysis was performed based on previous work by Engel et al. (2019) for differences of means and effect size estimation [33].

For microstates analysis, the Bayes factor (BF) was estimated using the JASP toolbox with a one-way ANOVA with a fixed factor for cohort and a dependent variable which contained the parameters from the corresponding micro state. Briefly, using prior model probabilities, posterior model probabilities, and the Bayesian factor, which shows the change from prior to posterior model odds (BF), were calculated. As described by Wagenmakers et al., (2011) Bayes factors above 3 were considered to be significant, Bayes factors above 10 to be highly significant. Bayes factors above 100 were considered to show highest significance and were marked with *** [34]. For group differences and effect size see also Supplementary Table. 2.

## Results

The *PRG-1*^*R345T*^ SNP was found to occur in the population cohort studies SHIP and SHIP-Trend [14] at a heterozygous variant frequency of 0.86% and in the Gutenberg Brain Study (GBS) at a frequency of 0,91% (see also Table S1). Analysis of three large human population cohorts (SHIP, SHIP-Trend and the GBS), resulted in identification and analysis of heterozygous *PRG-1*^*R345T/WT*^ human subjects.

### *PRG-1*^*R345T/WT*^ alters E/I balance and threat processing in human brain

**Fig. 1:**
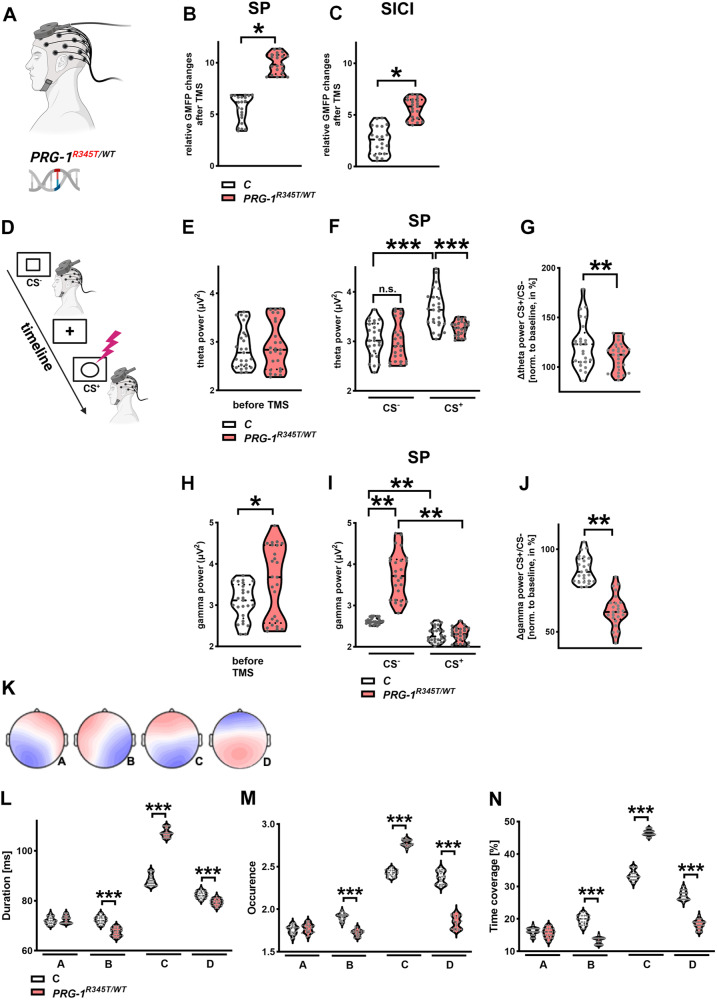
Cortical network excitability shows significant dynamic alterations in *PRG-1*^*R345T/WT*^ mutation carriers. **A** Experimental design for the assessment of excitability changes and E/I balance shifts in the frontal cortex of *PRG-1*^*R345T/WT*^ carriers using high-density EEG and TMS stimulation of the dmPFC. **B** SP: Global mean field potential (GMFP) over frontal cortex after single TMS-pulses (SP) over the dorsomedial prefrontal cortex (dmPFC) was significantly increased in *PRG-1*^*R345T/WT*^ carriers (*n* = 25) when compared to age and sex matched control subjects (*n* = 25). **C SICI**: After inhibitory Double TMS-pulses (SICI) over the dm PFC, GMPF over the frontal cortex in *PRG-1*^*R345T/WT*^ carriers (*n* = 25) was lower when compared to values after single pulse, however, it was significantly higher than in control subjects (*n* = 25). **D** Experimental design of the instructed fear paradigm. Unconditioned/safety Stimulus (CS^-^) or a conditioned stimulus (CS^+^), which in 1/3rd of presentations was accompanied by an electric shock, were presented for 5 s following an image of a fixation cross (5–6 s). Single-pulse TMS was applied on the dmPFC 1 s after presentation of each stimulus. **E** Global theta power over frontal cortex at baseline was not altered in *PRG-1*^*R345T/WT*^ carriers (*n* = 25) when compared to matched control subjects (*n* = 25). **F** Theta power over the frontal cortex was not different between control subjects (*n* = 25) and *PRG-1*^*R345T/WT*^ carriers (*n* = 25) following CS^-^ presentation and single pulse TMS over dmPFC. However, following CS^+^ presentation and SP TMS, global theta power was significantly increased in control subjects, while a significant lower theta power was observed in *PRG-1*^*R345T/WT*^ carriers under same conditions. **G** Theta power ratio of GMFP changes following SP with prior CS^+^ or CS^-^ presentation (normalized to baseline theta power) displayed significantly reduced increase in *PRG-1*^*R345T/WT*^ carriers (*n* = 25) when compared to control subjects (*n* = 25) under same conditions. **H** Global gamma power over the frontal cortex at baseline was significantly increased in *PRG-1*^*R345T/WT*^ carriers (*n* = 25) when compared to matched controls (*n* = 25). **I** After CS^-^, TMS over dmPFC resulted in significantly higher gamma power over the frontal cortex in *PRG-1*^*R345T/WT*^ carriers (*n* = 25) when compared to control subjects (*n* = 25). However, after presentation of a conditioned stimulus (CS^+^), gamma power was supressed and significantly lower in *PRG-1*^*R345T/WT*^ carriers when compared to CS^-^ conditions and was not different to control subjects. **J** Ratio of gamma power following SP with CS^+^ to SP with CS^-^ (both normalized to baseline gamma power) showed higher supression of gamma power in *PRG-1*^*R345T/WT*^ carriers (*n* = 25) following CS^+^ when compared to control human subjects (*n* = 25) under same conditions. Data in A-J are represented as violin plots covering all individual data points. Median, lower and upper quartiles are shown by dotted lines (* and ** show group differences of * >80% or **>90%, Bayesian analysis). **K**. Spatial configuration of the four microstate classes (**A**, **B**, **C**, **D**) according to EEG analyses. **L**–**N** EEG-analyses of the temporal microstates parameters for mean duration (shown in **L**), occurrence (shown in **M**) and time coverage (shown in **N**) revealed significant increases in microstate (**C**) and reduction in microstates (**B**, **D**) in *PRG-1*^*R345T/WT*^ carriers (*n* = 25) when compared to control subjects (*n* = 25). Age and gender were calculated as covariates. Analyses were performed using Bayesian one-way ANOVA. *** represent highest significance for Bayes factors >100. Data is shown as violin plots covering all individual data points. Median, lower and upper quartiles are shown by dotted line.

Although E/I changes in cortical networks play an important role for mental disorders, translational evidence from mice to humans is missing. We conducted EEG-recordings, which allow for high temporal resolution of oscillatory brain frequencies in the millisecond range. In order to assess for alterations in cortical excitability, which we hypothesized in *PRG-1*^*R345T*^ expressing humans, we assessed global mean field power (GMFP) changes in cortical network excitability following transcranial magnetic stimulations (TMS) over the dorsomedial prefrontal cortex (dmPFC) (Fig. 1A). Single TMS pulses (SP) over the dmPFC under resting state conditions significantly increased GMFP over the frontal cortex in *PRG-1*^*R345T/WT*^ carriers compared to control subjects (Fig. 1B). This difference was smaller but still significant for inhibitory double TMS pulses (Fig. 1C). In line with data showing higher neuronal excitability in *Prg-1*^*R346T*^ mice [8], our data suggest a shifted E/I balance and higher cortical network excitability in human *PRG-1*^*R345T/WT*^ carriers.

Our previous data show that excitability regulation of cortical networks is important for threat processing. Here, increased network activity displayed highest event-related activity in the dmPFC while TMS pulses increased cortical excitability enhancing these responses [35–38]. Analyzes of conditional fear responses have a high translational potential [39]. We therefore analyzed functional effects of this E/I-shift in our established threat processing paradigm using a conditioned fear stimulus (experimental setting depicted in Fig. 1D) [35–38]. Since physiological responses to threat induce synchronized oscillations in cortical networks in particular in the dmPFC, hippocampus, and amygdala, the applied threat paradigm allows to assess excitability shifts during active processing in these networks [35–37]. Presentation of a conditioned stimulus (CS^+^) after baseline recordings induced an increase of theta power in control subjects (Fig. 1E, F), which was reported to correlate with improved stress coping behavior [35, 37]. In contrast, this response over the dmPFC was significantly reduced in *PRG-1*^*R345T/WT*^ subjects in absolute and relative values (Fig. 1F, G), indicating a deficit in cortical threat processing.

Gamma power, which reflects local neuronal activity [40], was increased in the frontal lobe in *PRG-1*^*R435T/WT*^ carriers under baseline conditions pointing to higher network excitability and shifted E/I balance (Fig. 1H). After application of a single TMS pulse (SP TMS) following a conditioned safety stimulus (CS^-^), gamma power over the dmPFC was significantly higher in *PRG-1*^*R435T/WT*^ carriers when compared to control subjects (Fig. 1I, left). However, when a TMS pulse was applied following a conditioned fear stimulus (CS^+^), gamma power was significantly decreased and was not different to controls under same condition (Fig. 1I, right). Since SP TMS and CS^+^ presentation profoundly altered gamma power, we performed analyses of SP TMS gamma normalized to pre-TMS baseline finding a significant decrease in SP TMS gamma power in the ratio of CS^+^/CS^-^ presentation in *PRG-1*^*R345T/WT*^ carriers (Fig. 1J). These changes correlate to reports showing decreased gamma oscillations in response to TMS in patients with neuropsychiatric disorders like Alzheimer’s disease (AD [41]) or Schizophrenia (SZ [42]).

### Altered E/I-balance affects resting state EEG microstates

In order to better understand the effects of altered E/I balance and increased network excitability on global cortical network changes, we analyzed resting state EEG microstates. Analyses of resting state EEG revealed highly reproducible scalp potential topographies with a duration of around 100 ms, which are described as microstates and are separated in four classes labeled A,B,C and D (Fig. 1K) [43]. Detailed microstate analysis showed a significant increase of class C in all analyzed categories (duration, occurrence, time coverage) and a decrease in class D in *PRG-1*^*R345T/WT*^ mutation carriers (Fig. 1L–N). These data suggest that E/I changes affect cortical networks and EEG microstates and may represent an intermediate phenotype for neuropsychiatric disorders as previously demonstrated in SZ patients and in their unaffected siblings [44].

### Human *PRG-1*^*R345T/WT*^ carriers and *Prg-1*^*R346T/WT*^ mice display a comparable phenotype

**Fig. 2:**
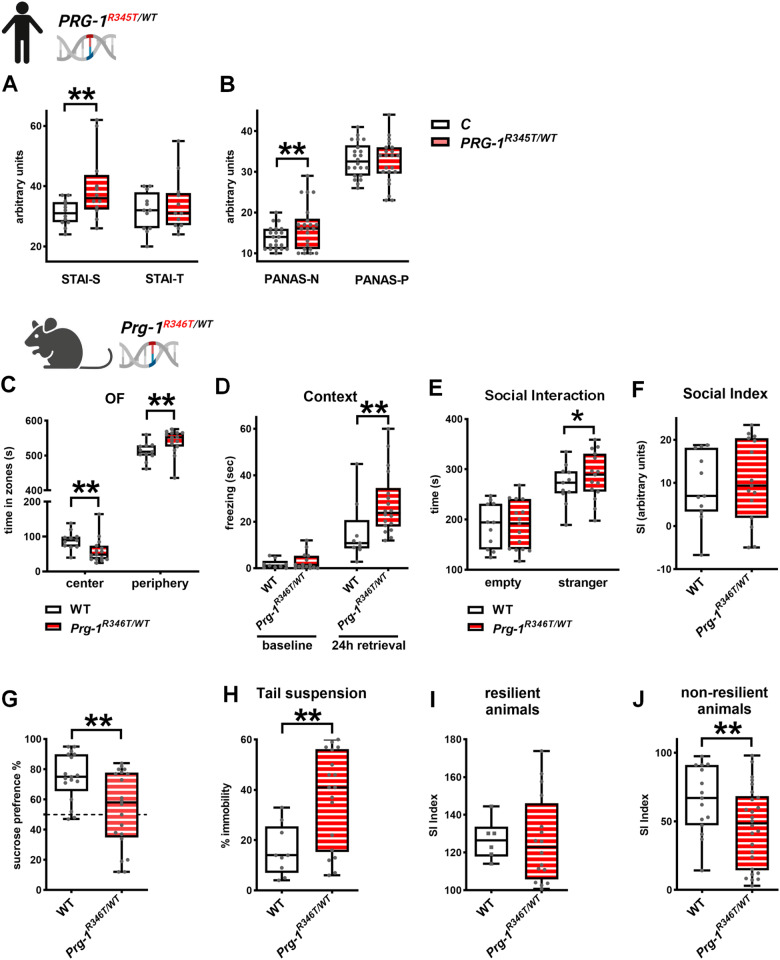
*PRG-1*^*R345T/WT*^ SNP results in a phenotype consisting of mild levels of anxiety, depression and reduced stress coping in humans and mice. **A** Human *PRG-1*^*R345T/WT*^ carriers displayed higher anxiety levels of state anxiety (STAI-S) but no difference in trait anxiety (STAI-T) when compared to control subjects as assessed by the State-Trait-Anxiety Inventory (STAI) (*n* = 12 control subjects and 12 *PRG-1*^*R345T/WT*^ carriers, Bayesian analysis). **B** In the positive and negative affect schedule (PANAS), *PRG-1*^*R345T/WT*^ carriers displayed higher levels for negative affects (PANAS-N) but no difference for positive affects (PANAS-P) when compared to control matched subjects (*n* = 21 control subjects and 22 *PRG-1*^*R345T/WT*^ carriers for PANAS-N and 22 control subjects and 22 *PRG-1*^*R345T/WT*^ carriers for PANAS-P, Bayesian analysis). **C** *Prg-1*^*R346T/WT*^ mice display reduced stay in the center of a novel open field (OF) arena and spent accordingly more time in its periphery when compared to WT litters (*n* = 11 WT mice, 14 *Prg-1*^*R346T/WT*^ mice, Bayesian analysis). **D** Fear conditioning revealed no significant difference at baseline but higher freezing 24 h after foot shock when animals were returned to the place where they previously received foot shocks (context). (*n* = 9 WT mice for baseline and after 24 h retrieval and *n* = 15 *Prg-1*^*R346T/WT*^ mice for baseline and 18 *Prg-1*^*R346T/WT*^ mice after 24 h retrieval; Bayesian analysis). **E**, **F** Social interaction and social interaction (SI) index were preserved in *Prg-1*^*R346T/WT*^ expressing mice. (*n* = 11 WT mice, 15 *Prg-1*^*R346T/WT*^ mice, Bayesian analysis). **G** Sucrose preference was significantly reduced in *Prg-1*^*R346T/WT*^ mice when compared to WT litters (*n* = 17 WT mice and *n* = 20 *Prg-1*^*R346T/WT*^ mice, Bayesian analysis). **H** Immobility in the tail suspension test (TST) following acute restrain stress was significantly increased in *Prg-1*^*R346T/WT*^ mice when compared to WT litters (*n* = 9 WT mice and *n* = 18 *Prg-1*^*R346T/WT*^ mice, Bayesian analysis). **I**, **J** Analysis of mice following chronic social defeat stress revealed no difference in social interaction index between *Prg-1*^*R346T/WT*^ mice and their WT litters in the group of resilient animals (defined by an SI index over 100). However, in the group of non-resilient animals (SI index below 100) *Prg-1*^*R346T/WT*^ mice displayed significantly reduced SI (*n* = 15 non-resilient WT mice and 26 non-resilient *Prg-1*^*R346T/WT*^ mice; *n* = 6 resilient WT mice, and 16 resilient *Prg-1*^*R346T/WT*^ mice; Bayesian analysis). Data are represented as box plots with whiskers covering all individual data points. Median is shown by solid line. (* and ** show group differences of * >80% or **>90%, Bayesian analysis).

Since decreased theta power following presentation of a painful conditioned stimulus points to an altered fear processing, we assessed anxiety states and emotional states of human *PRG-1*^*R345T/WT*^ mutation carriers. Here, *PRG-1*^*R345T/WT*^ mutation carriers displayed higher state anxiety (assessed in the State-Trait Anxiety Inventory [STAI]) suggesting a higher state arousal while no change in trait anxiety was observed (Fig. 2A). Assessment of positive and negative affect using the Positive and Negative Affect Schedule (PANAS P and N, respectively) revealed higher levels of negative emotions in *PRG-1*^*R345T/WT*^ mutation carriers (Fig. 2B).

Behavioral analysis of *Prg-1*^*R346T/WT*^ mice revealed an anxiety/depressive syndrome. Here, *Prg-1*^*R346T/WT*^ mice displayed lower stay in the center of the open field (Fig. 2C) and higher freezing in the same context 24 h after fear conditioning (Fig. 2D), both pointing to higher anxiety levels and altered fear processing. However, anxiety did not seem to be a trait in these mice, since social interaction was well preserved (Fig. 2E, F). Moreover, *Prg-1* ^*R346T /WT*^ mice showed lower sucrose preference, which reflects anhedonia and may be regarded as a readout for depressive behavior (Fig. 2G). Since findings in threat processing in human *PRG-1*^*R345T/WT*^ mutation carriers suggested reduced stress coping, we analyzed *Prg-1*^*R346T/WT*^ mice coping with acute and chronic stress. Following acute restrain stress, *Prg-1*^*R346T/WT*^ mice displayed higher immobility than control litters pointing to helpless behavior (Fig. 2H). Following chronic stress in the resident-intruder situation, animals assigned to the group of non-resilient animals (SI-index below 100, see Material and Methods) displayed significantly reduced social interaction (Fig. 2I, J). Both stress-related findings point to reduced stress coping in these mice corroborating the translational aspect of the *PRG-1*^*R345T*^ mutation in man and mice.

### E/I balance changes alters hippocampal network synchronization in humans and mice

**Fig. 3:**
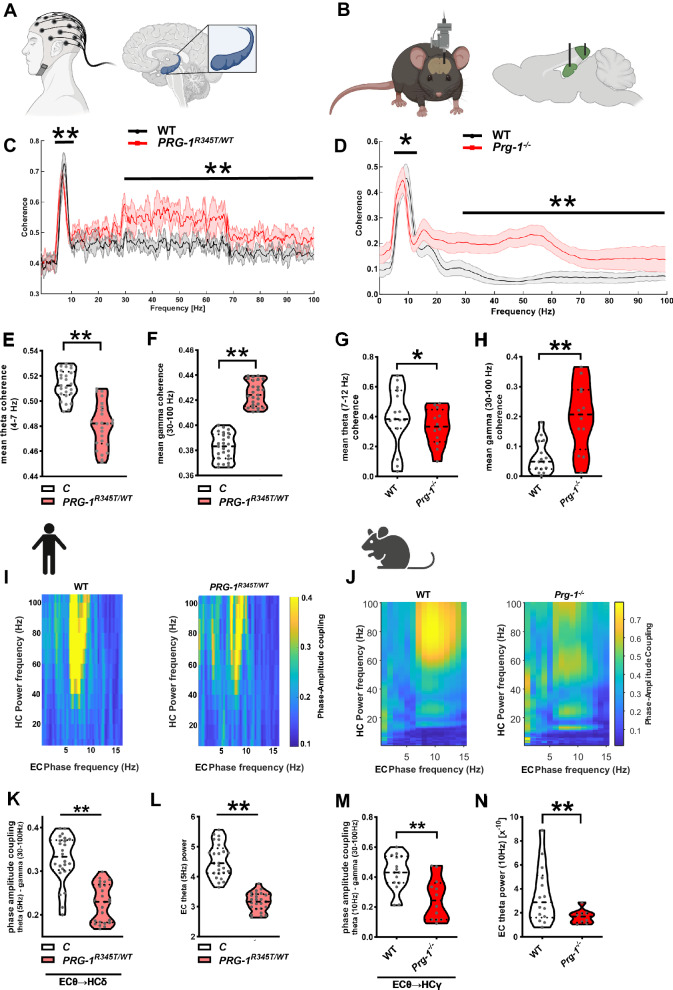
Analyzes on cortical network synchronization (coherence) revealed similar changes in *PRG-1*^*R345T/WT*^ human mutation carriers and in *Prg-1*^*−/−*^ mice. **A** Exemplary image of EEG coherence analysis in the human entorhinal-hippocampal network. **B** Exemplary image of invasive mouse in-vivo measures and synchronization analysis of the murine entorhinal cortex (EC) and hippocampus (HC). **C** *PRG-1*^*R345T/WT*^ human carriers revealed significant decrease in the theta range (4–8 Hz) and an increase in the gamma range (30–100 Hz) when compared to control human subjects (*n* = 25 control subjects, 25 *PRG-1*^*R345T/WT*^ carriers, Bayesian analysis). **D** In-vivo analysis of WT and PRG-1^−/−^ mice revealed significantly decreased theta coherence (7–12 Hz) and increased gamma coherence (30–100 Hz) when compared to WT litters. (*n* = 15 WT mice, 13 *Prg-1*^*−/−*^ mice, Bayesian analysis). **E**, **F** Theta coherence (4–8 Hz) between the EC and the HC was reduced in *PRG-1*^*R345T/WT*^ human carriers, while gamma coherence (30–100 Hz) was significantly increased (*n* = 25 control subjects, 25 *PRG-1*^*R345T/WT*^ carriers, Bayesian analysis). **G**, **H**. Theta coherence (7–12 Hz) in the entorhinal-hippocampal network of *Prg-1*^*−/−*^ mice was significantly decreased, while low and high gamma coherence (30–100 Hz) was significantly increased (*n* = 15 WT mice, 12 *Prg-1*^*−/−*^ mice, Bayesian analysis). **I** Representative phase-amplitude coupling (PAC) plots of EC theta and HC gamma. Left PAC plot shows high PAC levels of EC 5–8 Hz theta oscillation and hippocampal gamma power in the range of 40–100 Hz in a control human subject. Right PAC plots depicts reduced modulation of HC gamma power by EC theta frequency in a *PRG-1*^*R345T/WT*^ human mutation carrier. **J** Representative PAC plot of a wild type animal (left, WT) showing strong modulation of the hippocampal gamma power by entorhinal theta frequencies. Note the phase-amplitude correlation (yellow) showing highest values between 60 and 100 Hz for hippocampal gamma power modulated by 7–12 Hz entorhinal theta frequencies. Right plot displays representative PAC of a *Prg-1*^*−/−*^ mouse showing reduced phase-amplitude correlation (yellow). **K** PAC of entorhinal theta oscillation (5 Hz, EC θ) and hippocampal gamma power (30–100 Hz, HC γ) shows reduced PAC in *PRG-1*^*R345T/WT*^ human mutation carriers when compared to matched control subjects (*n* = 25 control subjects, 25 *PRG-1*^*R345T/WT*^ carriers, Bayesian analysis). **L** EC theta power (5 Hz) was significantly decreased in *PRG-1*^*R345T/WT*^ human mutation carriers when compared to control subjects (*n* = 25 control subjects, 25 *PRG-1*^*R345T/WT*^ carriers, Bayesian analysis). **M** PAC of EC theta oscillation (10 Hz, EC θ) and hippocampal gamma power (30–100 Hz, EC γ) shows reduced coupling in *Prg-1*^*−/−*^ mice when compared to their wild type litters (*n* = 15 wild type mice and 12 *Prg-1*^*−/−*^ mice, Bayesian analysis). **N** EC theta power (10 Hz) was significantly reduced in *Prg-1*^*−/−*^ animals (*n* = 14 WT and 10 *Prg-1*^*−/−*^). **E**–**H, K**–**N**. Data are represented as box plots with whiskers covering all individual data points. Median, lower, and upper quartiles are indicated by dotted line. (* and ** show group differences of * >80% or **>90%, Bayesian analysis).

The hippocampal formation displays highest levels of PRG-1 expression in the brain [45], hence this region is of specific interest when it comes to PRG-1-related alterations of neural functioning. Analyses of the hippocampal networks are supposed to reflect the effect of synaptic lipid mediated changes induced, e.g., by *PRG-1*^*R345T*^ loss-of-function most clearly. Using a 64-channel resting state EEG with deep source localization modeled by the aid of 3 T MRI data [46] (Fig. 3A), we assessed network dynamics in the entorhinal-hippocampal circuitry in human *PRG-1*^*R345T/WT*^ mutation carriers and compared it to in-vivo measures from the *Prg-1* deficient animal model (Fig. 3B). Power analysis of hippocampal theta and gamma oscillations in *PRG-1*^*R345T/WT*^ mutation carriers and in *Prg-1*^*−/−*^ mice revealed increased theta power and lower theta peak as well as higher gamma power both in humans and mice (Fig. S2A–E, G).

Coherence analysis between the entorhinal cortex (EC) and the hippocampus in *PRG-1*^*R345T/WT*^ human mutation carriers showed a reduced coherence in the theta range, lower theta peak coherence, and an increase in low and high gamma oscillations (Fig. 3C, E, F and Fig. S2F). This was in line with invasive hippocampal and entorhinal in-vivo measurements in freely behaving *Prg-1*^*−/−*^ mice, where we detected reduced theta coherence, lower theta peak coherence, and higher gamma coherence (Fig. 3D, G, H, and Fig. S2H). Since reduced coherence in the theta range reflects reduction in long-range communication, these data are in line with reduced connectivity between the EC and the hippocampus as shown in *Prg-1*^*−/−*^ animals. Here, using stereological methods, we could show that higher neuronal activity present in *Prg-1* deficient mice resulted in reduced axon numbers in the performant path, which connects the EC to the hippocampus [47]. Increased low and high gamma coherence, as present in human *PRG-1*^*R345T/WT*^ mutation carriers (Fig. 3F) and in *Prg-1*^*−/−*^ animals (Fig. 3H), reflects higher regional excitability and a shift in local E/I balance. Changes in the gamma range were typically found in patients suffering of psychiatric disorders as well as in their healthy relatives [48, 49].

### Synaptic lipid-mediated E/I balance regulates entorhinal-hippocampal phase-amplitude coupling

Physiological correlates of information processing, including memory formation, was described to be mediated via frequency modulation between different brain regions (so-called cross-frequency coupling or phase-amplitude coupling), which is regarded as a correlate of information transmission between two brain regions [50]. Hereby, theta frequencies in one brain region modulate higher frequencies in the gamma range of another region, and this modulation is favored by higher theta power [51]. Phase-amplitude coupling (PAC) was reported to be involved in hippocampal memory formation in humans [52]. In order to understand the effects of altered E/I balance and altered coherence on information transfer, we analyzed PAC in the entorhinal-hippocampal network. We detected reduced PAC between entorhinal theta (5 Hz) and hippocampal gamma (30–100 Hz) oscillations in human *PRG-1*^*R345T/WT*^ mutation carriers (exemplarily shown in Fig. 3I and quantified in Fig. 3K). This was in line with lower theta (5 Hz) power in the entorhinal cortex (EC) in human *PRG-1*^*R345T/WT*^ mutation carriers (Fig. 3L). This finding was specific and unidirectional since hippocampal theta (5 Hz) modulation of entorhinal gamma frequencies (30–100 Hz) was not altered (Supplementary Fig. S2I).

Translational analysis in *Prg-1* deficient mice revealed a significant decrease of PAC between the highly interconnected layer II/III of the entorhinal cortex and the hippocampal CA1 pyramidal cells (exemplarily shown in Fig. 3J, quantification shown in Fig. 3M). In line with human data, *Prg-1* deficient mice displayed lower theta power in entorhinal layer II/III neurons (Fig. 3N) and showed an unaffected PAC when analyzing hippocampal theta (10 Hz) modulation of entorhinal gamma oscillations (Fig. S2J).

### Intervention into synaptic lipid signaling by ATX-inhibition restores coherence and PAC

**Fig. 4:**
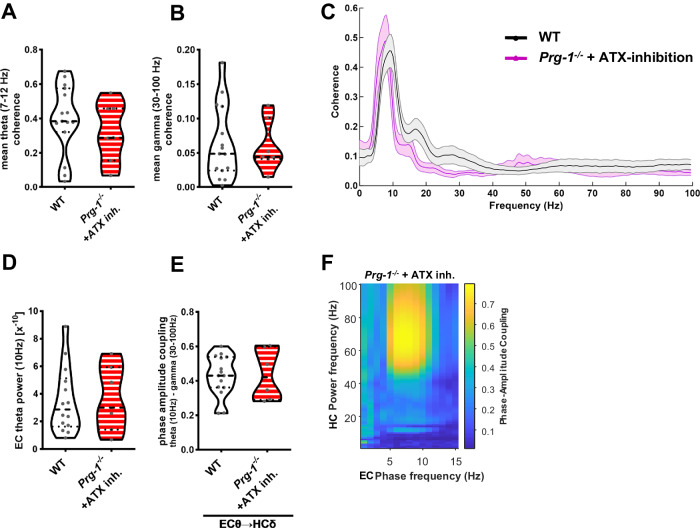
ATX inhibition restores entorhinal-hippocampal coherence and PAC to control values. **A** Inhibition of the LPA-synthesizing molecule ATX by PF8380 (ATX inh.) increased EC-HC theta coherence (7–12 Hz) in *Prg-1*^*−/−*^ mice to WT levels (*n* = 15 WT mice, 7 *Prg-1*^*−/−*^ + ATX inh. mice, Bayesian analysis). **B** Low and high gamma coherence (30–100 Hz) in the entorhinal-hippocampal network in *Prg-1*^*−/−*^ mice was reduced to WT levels following ATX inhibition (*n* = 15 WT mice and 7 *Prg-1*^*−/−*^ + ATX inh. mice, Bayesian analysis). **C** Coherence spectrum of WT and *Prg-1*^*−/−*^ mice following ATX-inhibition shows restored coherence to WT levels (*n* = 15 WT mice, 7 *Prg-1*^*−/−*^ + ATX inh. mice, Bayesian analysis). **D** EC theta power (10 Hz) in *Prg-1*^*−/−*^ animals was increased to WT levels following ATX-inhibition by PF8380 (*n* = 14 WT, 7 *Prg-1*^*−/−*^ + PF8380, Bayesian analysis). **E** PAC of EC theta oscillation (10 Hz, EC θ) and hippocampal gamma power (30–100 Hz, EC γ) in *Prg-1*^*−/−*^ mice increased back to wild type levels following inhibition of the LPA-synthesizing enzyme ATX (ATX inh.) (*n* = 15 wild type mice, 7 *Prg-1*^*−/−*^ + ATX inh. mice, Bayesian analysis). **F** Representative PAC of a *Prg-1*^*−/−*^ animal following ATX-inhibition. Note the restored PAC between the entorhinal theta frequency (10 Hz) and the hippocampal gamma power (30–100 Hz) in the *Prg-1*^*−/−*^ animal after ATX inhibition. **A**, **B**, **D**, **E** Data are represented as violin plots covering all individual data points. Median, lower and upper quartiles are shown by dotted linie (* and ** show group differences of * >80% or **>90% for Bayesian analysis).

We have previously shown that inhibition of the main LPA-synthesizing enzyme autotaxin (ATX) reduced neuronal excitability as well as E/I balance in cortical networks [6]. Therefore, we tested whether ATX-inhibition by the specific, small inhibitory molecule PF8380 [6, 53], is able to restore consequences of shifted E/I balance in the synchronization of the entorhinal cortex and the hippocampus in *Prg-1* deficient mice. Following a dose-response curve for PF8380 on ATX-inhibition, we found an IC50 inhibition at 4 nM and an IC90 inhibition at 17,2 nM (Suppl. Fig. S2K). In our previous findings, following i.p. application of 30 mg/Kg bodyweight, we detected a CSF concentration of 360–420 nM, which, in line with the above presented inhibtion data, resulted in a thorough ATX-inhibition [6]. Using the concentration of 30 mg/Kg bodyweight in our in-vivo electrophysiological experiments, theta coherence (7–12 Hz) and gamma coherence (30-100 Hz) in the entorhinal-hippocampal circuit were restored back to WT levels (Fig. 4A–C). To understand the functional effects of restored E/I balance, we interrogated the effect of ATX-inhibition on entorhinal-hippocampal PAC. ATX-inhibition increased EC theta power back to wild type levels (Fig. 4D) resulting in increased PAC comparable to that measured in WT (Fig. 4E, F).

In sum, our data revealed that increased synaptic lipid signaling, as induced by the *PRG-1*^*R345T*^ SNP, results in a shift in E/I balance leading to increased intraregional cortical network synchronization, which was detected in *Prg-1* deficient mice in a (back)-translational approach. Since lower theta and higher gamma coherence as well as PAC in the entorhinal-hippocampal network were restored to wild type levels by ATX-inhibition, the comparable findings in men and mice point to a high translational relevance of pharmacological ATX-inhibition.

### *PRG-1*^*R345T/WT*^ carriers show unaltered attention but altered motor performance

Our previous data suggest lower memory performance [54] and a deficit in sensorimotor integration in *Prg-1* deficient mice on the one side [5] but a higher motor performance in these animals on the other side [55]. To assess the phenotype of altered synaptic lipid signaling mediated changes in E/I balance in humans, we performed a neuropsychological assessment of human *PRG-1*^*R345T/WT*^ carriers and of their age and sex-matched controls (for details see Table 1). Basic attention as assessed by the alertness task in the test for attentional performance (TAP) was unaltered in *PRG-1*^*R345T/WT*^ mutation carriers. This is important to note, since unaltered attention is a prerequisite and potential bias for memory formation (see below). However, in the TAP flexibility task, where subjects had to select between numbers and letters by left or right button press, *PRG-1*^*R345T/WT*^ mutation carriers displayed significantly shorter motor reaction times (RT) and less reaction time variability (RTV) compared to controls. *PRG-1*^*R345T/WT*^ carriers also responded faster to auditory stimuli but not to visual stimuli. However, when it came to motor flexibility and subjects had to change hands, *PRG-1*^*R345T/WT*^ carriers showed no difference to control subjects (RT changing hands and RTV changing hands, Table 1). These data suggest that synaptic-lipid induced E/I balance shift leading to increased cortical excitability facilitates faster motor responses in *PRG-1*^*R345T/WT*^ carriers. However, higher cortical excitability may impede integration of sensory inputs when task complexity (i.e., changing hands) is increasing. Latter data are in line with findings in *Prg-1*^*−/−*^ mice showing higher stereotype motor performance e.g., on the rotarod [55] but lower motor performance on a narrowing beam where integration of sensory inputs is required for motor performance [5]. Higher locomotion, as present in *Prg-1*^*−/−*^ mice, was described as a phenotype of psychiatric disorders [56] and was show to be normalized by inhibition of synaptic lipid signaling in an animal model [6, 55]. Our data support the idea that synaptic lipid-related E/I-shifts and cortical network hyperexcitability may affect motor control in psychiatric disorders.

### *PRG-1*^*R345T/WT*^ carriers display deficits in memory formation and memory-related brain networks

**Fig. 5:**
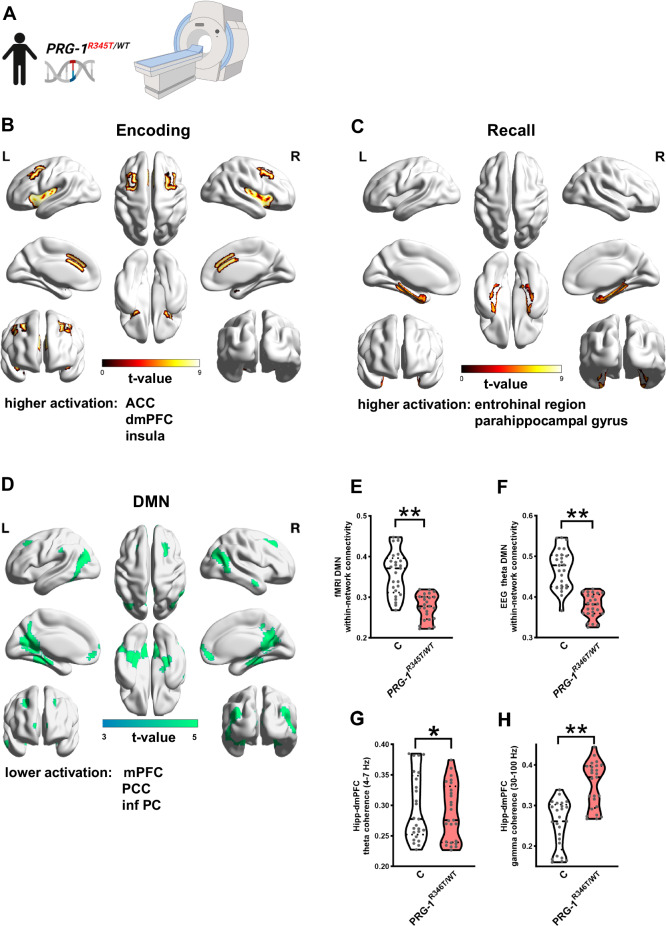
Functional imaging in *PRG-1*^*R345T/WT*^ mutation carriers revealed higher brain activation in an episodic memory paradigm and reduced DMN connectivity. **A** Experimental paradigm using fMRI. Human control subjects and *PRG-1*^*R345T/WT*^ carriers were subjected to an episodic memory task under fMRI conditions. **B** Regional brain activation during encoding in an episodic memory test shows higher bilateral activation in the anterior cingulate gyrus (ACC), in the dorsomedial prefrontal cortex (dmPFC) as well as in the insula (In) in *PRG-1*^*R345T/WT*^ human mutation carriers when compared to control subjects. **C** Regional brain activation during recall was significantly higher in *PRG-1*^*R345T/WT*^ mutation carriers in both hemispheres in the entorhinal region (EC) and in the parahippocampal gyrus (PHC). t-values for higher activation are represented by color bar in (**B**, **C**). **D** Default mode network (DMN) analysis of fMRI data during episodic memory test revealed lower activation of medial prefrontal cortex (mPFC), posterior cingulate cortex (PCC) and inferior parietal cortex (inf PC) in *PRG-1*^*R345T/WT*^ human mutation carriers when compared to matched control subjects. t-values for deactivation are represented by color bar. **E** fMRI network connectivity analyses within the DMN (within-network connectivity) showed lower connectivity of mPFC, PCC and inf PC. **F** EEG theta connectivity analyses within the DMN (within-network connectivity) revealed lower connectivity of mPFC, PCC, and inf PC. **G**, **H**. Resting state coherence in the prefrontal-hippocampal loop (dorsomedial prefrontal cortex, dmPFC) displayed reduction in the theta range and an increase in gamma range in *PRG-1*^*R345T/WT*^ human carriers when compared to matched controls All above mentioned analyses were performed in *n* = 25 control subjects, 25 *PRG-1*^*R345T/WT*^ carriers. Data are represented as violin plots covering all individual data points. Median, lower and upper quartiles are shown by dotted linie (* and ** show group differences of * >80% or **>90% for Bayesian analysis).

During assessment of cognitive performance, *PRG-1*^*R345T/WT*^ human mutation carriers displayed mild deficits in the Rey-Osterrieth Figure Test (ROF) and in the verbal learning memory test (VLMT; semantic memory). Here, mutation carriers displayed lower levels in delayed recall in the ROF suggesting reduction in visuospatial memory and in temporal lobe function [19]. In the VLMT, *PRG-1*^*R345T/WT*^ carriers showed a significantly lower performance at an early stage of memory formation. However, five learning rounds improved this encoding deficit, but in sum *PRG-1*^*R345T/WT*^ human mutation carriers displayed a significantly lower overall performance (Table 1). These findings are in line with previous findings showing that *Prg-1* deficient mice displayed impaired memory formation [54]. In order to better understand the role of *PRG-1*^*R345T*^-related E/I-shift in memory formation, we performed a well-established fMRI-based episodic memory (EM) task [57] (Fig. 5A), where memory formation depends on the entorhinal-hippocampal network. This test has been established for imaging genetics and allows to test cognitive processes involved in associative episodic memory formation, a process, which requires encoding, recall, and recognition of face-profession pairs [27]. The applied EM test has been used to describe the effect of genetic risk variants leading to potential intermediate phenotypes of psychiatric disorders [28, 58–60]. Here, fMRI analysis revealed higher brain activation in *PRG-1*^*R345T/WT*^ mutation carriers during encoding when compared to controls in the anterior cingulate cortex (ACC), in the dorsomedial prefrontal cortex (dmPFC), and in the insula (I) (Fig. 5B). During recall, *PRG-1*^*R345T/WT*^ mutation carriers displayed higher activity in the entorhinal region (EC) and in the parahippocampal gyrus (PHC) (Fig. 5C) while test performance of *PRG-1*^*R345T/WT*^ human mutation carriers in the episodic memory test was not altered when compared to controls (Table 1). These data suggest that the effects of a shift in E/I balance in terms of lower memory performance may be counterbalanced by higher prefrontal activation during encoding and higher entorhinal and parahippocampal activation during recall. Higher activation may reflect the need for a higher effort necessary to compensate for a reduced entorhinal-hippocampal memory processing in *PRG-1*^*R345T/WT*^ mutation carriers, which was not evident in the episodic memory test performance but in the more demanding VLMT (Table 1) as shown for other mutations leading to intermediate phenotypes of neuropsychiatric disorders [58].

### Altered E/I balance affects DMN connectivity in *PRG-1*^*R345T/WT*^ mutation carriers

Default mode network (DMN) comprises a collection of interconnected brain regions important for human cognition [61]. It is a network regarded to represent internally focused thought processes and was described to be, among other functions, important for episodic memory formation [62]. Reduced activity in the DMN was shown in Alzheimer´s disease (AD) patients and was suggested to be an early AD marker [63]. Using fMRI data, we analyzed data driven individualized DMN pattern during the episodic memory test. Here, we detected reduced activation in the medial prefrontal cortex (mPFC), posterior cingulate cortex (PCC), and in the inferior parietal cortex (inf. PC; Fig. 5D). We next estimated the directed connectivity between the identified regions (mPFC, PCC, and inf. PC) based on the group contrasted pattern within the DMN network (within-network connectivity) finding significantly reduced connectivity (Fig. 5E). To substantiate these findings, we analyzed resting state EEG data and assessed theta connectivity of the above-mentioned individualized pattern-based region of interest within the DMN. Theta based connectivity of the mPFC, PCC, and inf. PC within the DMN network (within-network connectivity) were lower in *PRG-1*^*R345T/WT*^ mutation carriers when compared to control subjects (Fig. 5F). Since both non-invasive brain measurements, EEG and fMRI, suggest lower connectivity we analyzed interindividual covariations to assess the relationship between these two methods. Here, we found a strong correlation between these two methods, both in control subjects (*r* = 0,67) as well as in *PRG-1*^*R345T/WT*^ mutation carriers (*r* = 0.72; Supplementary Fig. S3A–C). To further substantiate the deficits in the DMN, we analyzed EEG resting state coherence specifically in the hippocampal-dmPFC network in human *PRG-1*^*R345T/WT*^ mutation carriers. Here, we observed a decrease in theta coherence and an increase in gamma coherence in human *PRG-1*^*R345T/WT*^ mutation carriers when compared to control subjects (Fig. 5G, H). In so far, our data suggest, that altered E/I balance impairs well-balanced cortical network functionality and reduces connectivity of specific nodes impairing DMN integrative function.

## Discussion

In this translational study, we analyzed intermediate phenotypes of psychiatric disorders induced by a heterozygous loss-of-function single-nucleotide polymorphism (SNP) of the *PRG-1* gene (*PRG-1*^*R345T/WT*^) in human subjects and compared it to the effects induced by *Prg-1* gene deficiency (*Prg-1*^*−/−*^ and *Prg-1*^*R346T/WT*^) in mice. Intermediate phenotypes describe quantifiable measures of mild alterations, which were associated with specific genetic changes and which could be found as well in patients as in their unaffected relatives [64, 65]. Intermediate phenotypes were best described for mental disorders like schizophrenia and comprise electrophysiological measures like the P50 suppression test in an auditory double-klick paradigm and pre-pulse inhibition (PPI) [64], but also neuroimaging based connectivity analyses [66]. However, the concept of intermediate phenotypes is not restricted to schizophrenia, it also applies to other complex mental disorders like depression, attention deficit hyperactivity disorder or autism [65]. The described phenotypes in our work, which rely on increased E/I balance are also found in healthy individuals, are not dysfunctional per se, however, constitute intermediate phenotypes of psychiatric disorders as described by others [67]. Our in-depth analysis of synaptic lipid signaling on the cortical network level showed remarkable parallels between *PRG-1*^*R345T/WT*^ human mutation carriers and *Prg-1* deficient animal models regarding data processing and task response. Pharmacological intervention inhibiting lipid signaling in these mice normalized the intermediate phenotypes. Thus, dysregulated E/I balance at the synapse may translate into network and behavioral intermediate phenotypes of mental disorders in the human brain.

Investigation of *PRG-1*^*R345T/WT*^ human mutation carriers, although not displaying clinical diagnostic criteria for mental disorders, pointed to an altered fear processing and diminished stress coping. This was accompanied by higher state anxiety and higher mood negativity. In addition, elevated E/I balance enhanced motor reaction, which is in line with recent data suggesting that higher cortical excitability may boost motor function [68]. On a network level, *PRG-1*^*R345T/WT*^ human mutation carriers displayed significantly lower theta and increased gamma synchronization while phase-amplitude coupling was reduced in the entorhinal-hippocampal circuit. These findings, which suggest an altered neural network for memory formation were in line with a compensatory higher activity of memory-related regions in the temporal lobe, as shown in an fMRI correlated episodic memory test. On a behavioral level, these findings correlated with lower delayed recall performance in the Rey-Osterrieth Figure Test (ROF) and with lower memory performance in the verbal learning memory test (VLMT), which involves the entorhinal-hippocampal network.

*Prg-1*^*R346T/WT*^ mice, which express the homologous mutation, displayed higher anxiety levels, higher anhedonic disposition, and, most importantly, lower performance after acute restrain stress as well as decreased resilience following chronic stress. *Prg-1* deficient mice displayed similar changes like *PRG-1*^*R345T/WT*^ human mutation carriers in terms of theta and gamma power and coherence, but also regarding phase-amplitude coupling, which is regarded as the “neural code” for information transport and processing in the brain [69, 70].

In sum, our data suggest a three-level model: PRG-1 dysfunction at the synapse leads to (I) increased glutamatergic transmission [4], which (II) results in an increased excitatory network activity, and (III) in alterations of cognitive processing in the domains of fear conditioning and episodic memory formation as intermediate phenotypes of mental diseases (see also Suppl. Fig. S1 and Suppl. Table S2). Moreover, our data support the hypothesis that E/I balance alterations are of functional relevance impairing cortical information processing and demonstrating how compensation of these deficits may occur at the cortical network level. The high correlation of the human *PRG-1*^*R345T/WT*^ endophenotype to the corresponding *Prg-1* related phenotype in mice suggests that ATX-inhibition, which restored the E/I balance in *Prg-1* deficient mice to normal values, has high translational potential for a therapeutic intervention for cortical hyperexcitability-related mental disorders.

### Increased cortical network excitability alters dynamic response during stress coping

Extracranial TMS over the dorsomedial prefrontal cortex (dmPFC) allows for direct probing of cortical excitability [71, 72]. Single and double pulse TM stimulation over the frontal cortex in resting state EEG resulting in increased global field mean potentials (GFMP) showed effectiveness of TMS over dmPFC. Significantly increased GFMP following TMS points to higher dmPFC excitability in *PRG-1*^*R345T/WT*^ human carriers, which is in line with increased responses to TMS pulses reported in healthy volunteers following ketamine administration [73]. Cortical network reactivity was further analyzed in *PRG-1*^*R345T/WT*^ human carriers in an instructed fear paradigm, a task with high translational potential, and was combined with TMS over the dmPFC. In this paradigm, the dmPFC was reported to convey excitability regulation during threat processing following presentation of a painful conditioned stimulus (CS^+^) via increased excitability and synchronized activity with other structures of the fear network, which include the amygdala, the insular cortex, and the anterior cingulate cortex [35–38]. TMS application over the dmPFC 1 s after CS^+^ presentation prolonged this synchronized activity, which was detected by increased theta power in control subjects [35]. While control subjects, upon presentation of CS^+^ and TMS over the dmPFC, displayed a significant increase in frontal theta power, *PRG-1*^*R345T/WT*^ human carriers failed to show this response, indicating reduced long-range and short-range connectivity during fear stimulus CS^+^ processing [74]. Reduced or missing theta power increase was furthermore shown to be associated with reduced information flow and reduced threat processing, and points to lower stress coping abilities [35]. In terms of network excitability, these results suggest that preexisting higher excitability and shifted E/I balance, as present in *PRG-1*^*R345T/WT*^ human carriers, impaired dynamic modulation of cortical excitability and impeded an increase of cortical excitability and functional connectivity exemplarily shown during fear processing following CS^+^. This impaired modulation of cortical excitability is in line with previous data showing that increased glutamatergic transmission impairs short-term presynaptic plasticity as shown by reduced paired pulse ration (PPR) in *Prg-1* deficient mice [5]. However, on a behavioral level, reduced stress coping abilities in *PRG-1*^*R345T/WT*^ human carriers, were supported by higher values of state anxiety (STAI-S) and more negative emotions in the PANAS-N test. In a translational approach, *Prg-1*^*R346T/WT*^ animals displayed an increased fear response following fear conditioning, an anxiety/depressive phenotype, lower stress coping abilities, and lower resilience to chronic stress. In sum, these human and animal data suggest that changes in E/I-balance result in a susceptibility of *PRG-1*^*R345T*^ mutation carriers – although not presenting with a clinical disease.

### Synaptic lipids alter E/I balance and modulate brain oscillation

Despite the manifold increase in brain size, oscillations, as part of the “neural syntax” [70], remained constant among mammals [75]. Human *PRG-1*^*R345T/WT*^ carriers displayed reduced theta coherence (4–8 Hz) and increased coherence in the low (30–70 Hz) and high gamma range (70–100 Hz) in the entorhinal-hippocampal network, which were strikingly similar to findings in *Prg-1*^*−/−*^ mice. These data support shifted E/I-balance and increased regional neuronal excitability, which was reflected by increased coherence in the gamma range [76]. Gamma-band alterations were reported in psychosis and Schizophrenia (SZ) to the extent that an increase in gamma activity in resting state EEG was suggested to be a biomarker for glutamatergic dysfunction and an intermediate phenotype of SZ patients [77–79]. In line, our data show an increase in gamma coherence in the hippocampal-frontal connectivity in human *PRG-1*^*R345T/WT*^ mutation carriers, which is regarded as an intermediate phenotype for SZ [80].

Theta coherence has been associated with long-range connectivity in the brain and lower theta coherence with a reduction of it [69]. On a functional level, reduction in theta coherence as a measure for long-range connectivity was exemplarily shown for the hippocampal-frontal projection, a pathway that is affected in SZ [81]. Reduced hippocampal-frontal theta coherence in human *PRG-1*^*R345T/WT*^ mutation carriers in conjunction with the reduced sensory gating (measured by reduced p50 inhibition after an auditory double-klick paradigm [10]) can be regarded as an intermediate phenotype for psychiatric disorders [28, 57, 65].

Analysis of altered E/I-balance modulated brain oscillations, revealed significant changes in EEG microstates. These microstates are quasi-stable topographies with a duration of around 100 ms, which can be grouped in four classes (A-D). Microstate changes were reported in different neuropsychiatric disorders like mood and anxiety disorders [82], major depressive disorders [83], AD related dementia, and SZ [84]. The observed microstate changes (i.e., increase in C and decrease in D microstate parameters) in our study are largely comparable to changes observed in SZ patients and in their unaffected siblings suggesting that they may represent an intermediate phenotype for psychiatric disorders [44].

### E/I-balance shift in *PRG-1*^*R345T/WT*^ human carriers leads to deficits in neural memory circuitry

*PRG-1*^*R345T/WT*^ human carriers displayed affected memory in the Rey-Osterrieth Figure Test and in the semantic memory test (VLMT early phase, Table 1). In the episodic memory test, they showed a higher activation of memory-related circuits in the fMRI (Fig. 5B, C), pointing to a higher network effort in memory formation. These findings are well in line with the idea that higher activation in memory related regions represent a compensatory activity aiming to rescue cognitive performance. However, as shown by other groups, compensatory over-recruitment necessary for a similar performance like in control subjects may be restricted by a limited reserve capacity when performing a more demanding task i.e., in the VLMT, where *PRG-1*^*R345T/WT*^ human carriers displayed significantly reduced performance [58, 85]. Intriguingly, Erk et al. [59] found compensatory hyperactivity in the dlPFC in humans with subjective memory impairment, a risk state for developing Alzheimer’s disease, in the exactly same episodic task as used in our present study. These results brought us to the question in how far changes in excitability and subsequent E/I balance shifts may alter brain-wide circuits important for human cognition. Here, we assessed the default mode network (DMN), which is associated with key cognitive functions like self-reference, social cognition, episodic memory, and mind wandering [61]. Using episodic memory derived fMRI data, we found significant lower activation of specific DMN regions (mPFC, PCC, and inf PC), which were associated with episodic memory formation [62, 86]. Lower activation of these regions was supported by lower connectivity of those regions within the DMN network in the task-related fMRI and in resting-state EEG theta connectivity. Interestingly, both methods displayed significant correlation when analyzing interindividual covariations supporting the high predictive value of these methods.

Our data is in line with recent literature suggesting that DMN function in episodic memory acts as a whole with multiple representations engaging different DMN nodes rather than one specific region [87]. Furthermore, our data supports the idea that reduced activity of DMN nodes as observed in AD patients may impair episodic memory formation [63]. In sum, starting with higher glutamatergic release probabilities shown on the single neuron level, which result in higher cortical excitability and E/I change on the cortical network level, we found alterations in brain-wide network activation in specific regions of the DMN during a functional cognition test.

### E/I-balance changes reduce PAC which can be normalized by ATX-inhibition

The findings of higher compensatory activity in memory related regions during the episodic memory test and reduced connectivity within the DMN are supported by reduced phase-amplitude coupling (PAC) in the entorhinal-hippocampal system. PAC is regarded as a mechanism by which information is conveyed from a brain region to another and is measured by amplitude modulation of high frequency oscillations (gamma oscillations) by a low-frequency phase (theta oscillations). PAC is especially prominent in the neocortex and in the hippocampus [50]. Regarding its functional role, best evidence comes from learning experiments where the hippocampal PAC increased over time and correlated with performance improvement [88]. *PRG-1*^*R345T/WT*^ human carriers as well as *Prg-1* deficient mice, both displaying deficits in hippocampal memory formation, showed a significant reduction in PAC in the entorhinal-hippocampal network, which is well in line with reduced memory performance. Interestingly, ATX-inhibition in *Prg-1* deficient mice was able to normalize theta and gamma oscillations and to increase PAC in the entorhinal-hippocampal network back to control conditions.

Changes in E/I-balance have been for long time known to alter brain function [89], however, a direct link to a specific function has been missing. Our data show that E/I changes in cortical networks lead to deficits in information processing, which, however, due to compensatory brain activity resulted in mild cognitive deficits and decreased motor flexibility. In sum, we provide a detailed overview on cortical network changes in human subjects expressing a loss-of-PRG-1 function SNP (*PRG-1*^*R345T/WT*^) at glutamatergic synapses resulting in an altered E/I balance and in an intermediate phenotype for psychiatric disorders. We showed that neuropsychological changes had a rather low phenotypic penetrance and did not arrive at clinical diagnostic criteria for mental disease, whereas changes in cortical networks showed significant activity alterations. This indicates that circuits important for memory formation may exhibit enhanced activity in *PRG-1*^*R345T/WT*^ mutation carriers allowing for, however, limited functional compensation protecting from a full disease state. One limitation of the present study is the relatively small human subjects sample, which may result from the low frequency of the analyzed *PRG-1* SNP (0,87%). Furthermore, animal studies were restricted to male mice and were not aimed to detect sex differences.

E/I dysfunctions have been suggested to provide transdiagnostic explanations and to combine genetic and neurobiological evidence that exists within and between psychiatric conditions [90]. Due to missing clear molecular mechanisms underlying E/I dysfunction, this hypothesis has been hard to be proven. Using a thorough translational approach in combination with multimodal neural network analyses, we were able to pinpoint changes from genetics to synaptic changes leading to local neuronal network alteration as well as to global neural network changes including the cross-validation and causal intervention in an animal model. Hence, this study provides a mechanistic chain reaching from molecular to phenotypic alterations as one example of the long-sought causes of E/I imbalance [90].

The high man-to-mice correlation showed in our work, allowed us to take advantage of experimental therapeutic interventions possible in mice. We could show that inhibition of synaptic lipid signaling allowed for restoration of cortical networks to baseline levels. Since we found that inhibition of synaptic lipid signaling reduced cortical network hyperexcitability to normal values independent of the underlying cause [6], our current data suggest that pharmacological ATX-inhibition may be a new therapeutic concept for neuropsychiatric disorders associated with E/I disbalance.

## Supplementary information

Supplementary Material and Methods

Supplementary Figures 1–3

## Data Availability

All data associated with this paper are present in the paper or in the Supplementary Materials.

## Material availability

Materials used in this study will be available for the scientific community by contracting the corresponding authors and, when appropriate, completion of a material transfer agreement.
