# Supplementary material for: Altered cortical synaptic lipid signaling leads to intermediate phenotypes of mental disorders
Source: Mol Psychiatry. 2024 May 28;29(11):3537–52. doi: 10.1038/s41380-024-02598-2 (PMC11541086; doi:10.1038/s41380-024-02598-2)
Supplement: Supplementary file 1 — Supplementary Material and Methods [file 41380_2024_2598_MOESM1_ESM.docx]

**Supplementary Methods**

**Altered cortical synaptic lipid signaling leads to intermediate phenotypes of mental disorders**

Tüscher O., Muthuraman M., Horstmann JP. et al.,

**Human Studies**

**Participants (additional details)**

After being screened for inclusion and exclusion criteria in the studies’ databanks, participants were recruited over the phone and rechecked on site for anamnestic neurologic disease and psychiatric disorder using the MINI structured interview held by a mental healthcare professional ^1^. To ensure that results were not influenced by nicotine withdrawal, smoking ad libitum was allowed before each experiment ^2^.

**General neuropsychological assessment (additional details)**

In the TAP shared attention task, acoustic stimuli of two alternating high- and low- frequency tones of 450 and 1,070 Hz respectively were presented. Tones were presented with 1 s Interstimulus Interval (ISI) by speakers. Target stimuli were two repeatedly presented stimuli of identical pitch. The visual stimuli were 4 different symbols of which two were target stimuli. The visual stimuli were presented on a screen with 2 s ISI. Subjects were asked to equally pay attention to visual and auditory stimuli and to respond to target stimuli by button press ^3,4^. In the TAP flexibility task, subjects were presented a pair of two symbols, one being a number, the other a letter. They were asked to alternately select number or letter by left button press for the symbol presented on the left, and right button press for the symbol on the right. Position of letters and numbers switched in a pseudorandomized pattern throughout the task ^4^. With regard to the rerun of the alertness task at the very end of the session, repeated measures ANOVA correcting for site revealed no group differences to the first run at the very start for tonic alertness, phasic alertness or intrasubject differences for tonic-phasic alertness.

**Resting State EEG**

Continuous electroencephalography (EEG) was recorded for 5 minutes with eyes closed for resting state analysis. EEG data were recorded using a 64-channel EEG cap with passive silver/silver-chloride electrodes from EEG Recording Caps and Products GmbH, Breitbrunn, Germany (Easycap®) and recording equipment system from Brain Products GmbH, Gilching, Germany (BrainAmp DC Plus®). One set of equipment was used at both sites. Data were recorded relative to the FCz reference. The sampling rate of the analogue unfiltered data was 5000 Hz. Impedance at all recording electrodes was less than 10 kOhm.

**Instructed/conditioned fear task with concomitant dmPFC-TMS (additional details)**

In this experiment, the participants were asked to sit on a chair and to look at the screen. Following, painful electric stimuli were applied to the dorsal part of left hand using a surface electrode connected to a DS7A electrical stimulator (Digitimer). Individual pain ratings on a scale from 0 (no pain) to 10 (very unpleasant) were given by each individual. An intensity representing a pain level of 7 was used during the experiments. The instructed fear paradigm was developed using the Cogent toolbox (http://www.vislab.ucl.ac.uk/cogent_2000.php) in Matlab R2006b (The MathWorks). Participants were instructed that the screen appearance of a circle (CS+) is associated with a probability of 33% (randomized between 1- 5 seconds) of receiving the electric shock (US) of level 7; while screen appearance of a square (CS-) is not associated with any threat. Visual stimuli were presented pseudo-randomly on screen for 5 sec and the ITI was jittered between 4 and 6 sec. The experiment was divided in 3 sessions, where each session lasted around 5 minutes with 3 min breaks in between sessions. To evaluate the involvement of the dmPFC in threat processing and network dynamics, single TMS pulses over the right dmPFC, 1 sec after stimulus onset, were applied. The MNI (Montreal Neurological Institute) coordinate for the dmPFC ([10 12 58]) was obtained from a previous study and translated into the international 10-20-system (Gonzalez-Escamilla et al., 2018)^5,6^. Individual coordinates were determined using the corresponding MRI in SPM8 (http://www.fil.ion.ucl.ac.uk/spm). Location for TMS pulses delivery, coil position and orientation were controlled throughout the experiment using the 10-20-system by reference of the EEG cap. At the stimulation site the TMS coil was placed tangentially to the scalp surface and oriented in a medial to lateral position at a 45° angle away from the midline with the handle pointing backwards. TMS pulses were applied in biphasic pulse configuration using a figure-8 coil connected to Magstim Rapid^2^ (Magstim, UK). The intensity of TMS pulses was set to 110% RMT (resting motor threshold). RMT was calculated as the minimum stimulus intensity required eliciting motor evoked potentials of an amplitude of 50 µV in 5 out of 10 consecutive trials at rest (Groppa et al., 2012). The paradigm consisted of 100 stimuli (54 CS+, 36 CS-). The condition specific (CS-: no threat, CS+: threat) trials were considered and the trials in which shock was applied were removed from the analyses. Double pulses were applied using a 90% pulse followed by a 110% pulse with an interval of 4 msec in case of short intracortical inhibition (SICI) ^7^. A total of 100 TMS pulses were analyzed for each condition (Single Pulse vs SICI).

**EEG data preprocessing**

Initially, EEG data was re-referenced to the common grand average reference of all EEG channels and epoched from –2.0 to 4.0 s (0 - being the visual stimuli). These epoch trials were used for the purpose of filtering only, for all subsequent analyses the time interval for the epochs was –0.25 to 1.5 s. The preprocessing pipeline was adapted from the Fieldtrip toolbox explained detail in ^8^. For TMS-EEG data, a period of –5 to 20 ms relative to the TMS pulse was first cut out and excluded to remove the ringing artifact. The pre-ringing and post-ringing epochs were subject to FastICA to remove components representing the exponential decay artifact, residual muscle artifacts, eye blinks, eye movements, line noise and other muscle artifacts unrelated to TMS. On average for the experiment, 10 of 64 components (3 ± 1.6, mean ± SD) were rejected, 2-3 were related to the eye artifacts (2 ± 1.68), 1-2 related to line noise (1 ± 1.34) and 1-2 were related to muscle artifacts (1 ± 1.24). The residual muscle artifacts were visually inspected, removed and interpolated with the cubic interpolation method. A fourth-order Butterworth low-pass filter with a cut-off frequency of 200 Hz was applied to avoid aliasing.

**Analysis of brain activity**

The forward problem is the computation of the scalp potentials for a set of neural current sources. An established procedure was used by estimating the lead-field matrix with specified models for the brain; a volume conduction model with a finite-element method (FEM) was used (Wolters et al., 2007). For the forward modelling the surfaces of the compartments like the skin, skull, CSF, gray matter, and white matter extracted from the individual T1 MRI, and individual electrode locations were used. The forward modeling and the source analysis were done in FieldTrip (Oostenveld et al., 2011). The lead-field matrix (LFM) contains information about the geometry and conductivity of the model. The complete description of the solution for the forward problem has been described previously (Muthuraman et al., 2010, Muthuraman et al., 2012). A full description of the beamformer linear constrained minimum variance spatial filter is given elsewhere ^9,10^. The output of the beamformer at a voxel in the brain can be defined as a weighted sum of the output of all EEG channels. The weights determine the spatial filtering characteristics of the beamformer and are selected to increase the sensitivity to signals from a voxel and reduce the contributions of signals from (noise) sources at different locations. The frequency components and their linear interaction are represented as a cross-spectral density (CSD) matrix. In order to visualize power at a given frequency range, a linear transformation was used based on a constrained optimization problem, which acts as a spatial filter ^11^. The spatial filter assigned a specific value of power to each voxel. For a given source the beamformer weights for a location of interest are determined by the data covariance matrix and the LFM. A voxel size of 5 mm was used in this study, resulting in 6676 voxels covering the entire brain. The created source model was then interpolated on the brain regions defined according to the Harvard Oxford cortical-subcortical regions of interest (ROIs) defined in the MNI space. For each frequency band (theta and gamma) the activated voxels were selected by a within-subject surrogate analysis to define the significance level, which was then used to identify voxels in the regions as activated voxels. Once the brain region voxels were identified, their activity was extracted from the source space. In a further analysis, all the original source signals for each Harvard Oxford (HO) region ^12^ with several activated voxels were combined by estimating the second order spectra and employing a weighting scheme depending on the analyzed frequency range to form a pooled source signal estimate for each region separately for both stimulus (CS+, CS-).

**Structural and Functional magnetic resonance imaging**

First imaging was performed using a sagittal 3D T1-weighted magnetization prepared rapid gradient echo imaging (MP-RAGE) sequence (TE/TI/TR = 2.52/900/1900 ms, flip angle = 9°, field of view (FOV) = 256 × 256 × 192 mm^3^, matrix size = 256 × 256 × 192, voxel size = 1 × 1 × 1 mm^3^ BW = 170 Hz/pixel) and sagittal 3D T2-weighted fluid attenuated inversion recovery (FLAIR) sequence (TE/TI/TR = 388/1800/5000 ms, echo-train length = 848, FOV = 256 × 256 mm, matrix size = 256 × 256, slab thickness = 192 mm, voxel size = 1 × 1 × 1 mm). Tissue abnormalities were excluded by a specialized neuroradiologist. For the voxel-based morphometry (VBM) analysis, the VBM8 toolbox (<http://dbm.neuro.uni-jena.de/vbm/>) was used. We estimated the brain volumes based on the HO atlas.

For acquisition of functional magnetic resonance imaging (fMRI) data a Siemens Trio 3T was used in the Mainz site and a 3T Siemens Verio in Greifswald (Siemens Medical Solutions, Erlangen, Germany).

After these 10 minutes resting state measurement was obtained, in which participants would be asked to freely ruminate with their eyes open. For the resting state measurement, we chose a gradient echo (GE)-EPI sequence (TR = 3000 ms, TE = 30 ms, flip angle = 90°, field of view (FOV) = 192 × 192 mm², matrix size = 64 × 64, spatial in-plane resolution: 3 mm, 49 slices with a slice thickness of 2 mm and an inter-slice gap of 1 mm, readout bandwidth (BW) = 2232 Hz/pixel). A series of 200 volumes were acquired, resulting in scan duration of 10 min. At both sites, identical sequences and scanner protocols were used. Of all subject enrolled two were excluded from MRI measurements due to claustrophobia, resulting in a total of 25 R345T-allele carriers and 25 control subjects. Pre-processing of fMRI scans and time series extraction were performed using the statistical parametric mapping toolbox (SPM8; <http://www.fil.ion.ucl.ac.uk/spm>). For each subject, realignment was first performed to remove movement related artifacts (e.g. head motion) in fMRI time series. In order to realign, the first image from the recording was specified as the reference image and all subsequent images were realigned to it. Scans were then spatially normalized to align all the subjects’ specific MR sequences into the standard Montreal Neurological Institute (MNI) space. The scans were smoothed by convolving them with a Gaussian kernel of fixed width (full width half maximum 8 × 8 × 8 mm) to suppress noise and effects due to differences in anatomy ^13^.

After preprocessing, the time series extraction of all 113 Harvard Oxford atlas regions of interest (ROIs) was carried out using the CONN toolbox ^14^. To gain a conceptual and robust overview of functional connectivity changes in the entire brain in each region the time series were extracted according to the low frequency fluctuations amplitude (ALFF), ranging from 0.009–0.08 Hz ^13^. For connectivity matrix reconstruction we applied the same node definition as above. We extracted the individual volumes values for each ROI from the HO-Atlas for the construction of the structural correlation matrices. Similarly, the ALFF was used to construct the functional correlation matrix. Finally, the connectivity matrices were incorporated into the network analysis to describe the topological organization of structural and functional cortical networks in our subjects. In this study, we adopted two different analysis strategies in order to appropriately quantify the network topologies. For the functional MRI matrices, we calculated the network measures in every subject and included this data in the further network topological parameter analysis. For the structural volumetric connectivity, matrices were calculated for each group. Afterwards the network topological parameters were calculated for the group. In order to assess the network connectivity at different levels, we analyzed the most important measure of network topology, namely modularity. Brain networks can be separated into modules. Modules are densely interconnected nodes that have only sparse interconnections to other modules. Modularity is defined as the relation of the within-module and between-module connections ^15^. In this study, the modularity (Q) was calculated using the Newman algorithm ^16^.

**fMRI-Episodic Memory Task (Face-Profession association task) and DMN analysis**

Episodic memory tasks were performed during fMRI scanning. Thereby, participants had to complete 3 consecutive memory tasks (i.e., encoding, recall, and recognition of face-profession pairs) based on an established paradigm used in imaging genetics ^17,18^. The test had an overall duration of 13 minutes and is based on a paradigm previously described ^19^. During the encoding task, participants were presented 16 face-profession pairs and 24 head contours as control condition. Tests were performed with 4 blocks of 4 face-profession pairs and 4 blocks of 6 head contours each. Face-profession pairs were presented for 6 seconds each and head contours for 4 seconds each. In sum, each block lasted 24 seconds. Participants had to imagine the presented person acting in the given profession. Following this, participants were asked to state if the profession suited the person's face to induce deep encoding. In the control experiments, participants had to point to the larger ear of the depicted head contour. In an alternating sequence, 4 face-profession association blocks and 4 control blocks were presented twice to ensure successful encoding. In the recall phase, faces were presented again. Participants were asked, whether the depicted person had to complete apprenticeship or academic studies to execute the respective profession presented during the encoding stage. Hereby, participants had to state, by pressing a button, which qualification was correct. The stimulus duration and control condition were similar like in the encoding stage and blocks were presented only once. In the recognition testing, faces were presented together with 2 written professions, and participants were asked to indicate which profession was correct. The stimulus duration for recognition was 3 seconds. The control condition consisted of 4 blocks of 4 head contours each (for 3 seconds each). Thus, each recognition block lasted 12 seconds. In line to the recall stage, blocks were presented only once. Furthermore, to interrogate the effects of higher cortical excitability and altered E/I balance on the default mode network (DMN) during the episodic memory task, we assessed DMN connectivity and estimated activation of DMN regions in the fMRI during this task. DMN was defined based on the Schaefer atlas ^20^. Individual activation patterns were assessed for each group within the DMN. In a first step, we looked at the contrast between the PRG-1^R345T/WT^ and control subjects for the entire task blocks including encoding, recall and recognition. Here we found deactivation patterns, which are depicted as color-coded t-values. In a second step, data-driven pattern found within the DMN were defined as region of interest for connectivity analyses and were estimated correspondingly. Thirdly, in order to corroborate the connectivity with the fMRI data, we validated this data and calculated theta frequency band connectivity using EEG data ^13,21^.

**Animal studies**

**Mouse lines**

C57Bl/6J were obtained from Janvier, France. PRG-1^R346T^ and PRG-1^+/-^ male transgenic mice were generated as previously described and were genotyped accordingly ^22,23^.

***In-vivo*** **electrophysiological recordings** were performed as previously described ^24^. Briefly, anesthetized animals were chronically implanted with electrodes (WE3PT10.5F3, MicroProbes, Gaithersburg, MD, USA) in the medial entorhinal cortex (MEC; 3.1mm, lateral to the midline, 0.2mm anterior to the transverse sinus at an angle of 6° in the anterior-to-posterior direction in the sagittal plane) and in the hippocampal CA1-region (1.5 mm lateral to the midline, 1.5 mm anterior-to-posterior and 1.2 mm dorsal-to-ventral at an angle of 0°). Recording location was verified after animal perfusion in cresyl violet stained sagittal cut brain slices.

**Cross-frequency coupling in PRG-1^-/-^ mice**

The local field potential (LFP) signals from MEC and CA1 were convolved with a series of Morlet wavelets with center frequencies ranging from 1 to 100 Hz and a length of three cycles, resulting in a wavelet transform of the LFP signal. At each frequency we estimated the power and phase of each LFP signal as the absolute value and angle, respectively, of its wavelet transform. We then calculated how the power of the LFP signal from CA1 at frequency *y* varied as a function of the phase of the signal from MEC at frequency *x*. To this end, we divided the phases of the MEC signal at frequency *x* into 20 bins from -180 to +180 degrees and for each phase bin calculated the average power of the CA1 signal at frequency *y*, resulting in a vector *p* of average power values in each phase bin.

**Behavioral analyses**

**Open field (OF)**

Spontaneous activity in the OF was tested in a gray Perspex arena (40 x 40 x 40 cm illumination 120 lx) as described earlier ^25^. The mice were placed in the center and allowed to explore the OF for 10 min. The behavior was recorded by an overhead video camera and a personal computer (PC) equipped with “Ethovision XT 8.5” software (Noldus, Inc.) to calculate velocity, distance traveled, and time spent in central, or peripheral zones of the OF.

**Social interaction index (SI)**

Three-chamber sociability and social memory.

Sociability was evaluated as described earlier ^25^. The social testing arena was a rectangular, three-chambered box. Each chamber was 20 × 40 × 22 cm in size. Dividing walls were made from clear Plexiglas, with rectangular openings (35 × 35 mm) allowing access into each chamber. The test mouse was first placed in the middle chamber for 5 min of habituation. The openings into the lateral chambers were obstructed by plastic boxes during this habituation phase. After the habituation period, an unfamiliar WT male mouse (“stranger”) was placed in one of the lateral chambers. The location of stranger in the left vs. right side chamber was systematically alternated between trials. The stranger mouse was enclosed in a small (60 × 60 × 100 mm), rectangular wire cage, which allowed nose contact through the bars but prevented fighting. The animals serving as strangers had previously been habituated to placement in the small cage. An identical empty wire cage was placed in the opposite chamber. Both openings to the side chambers were then unblocked, and the subject mouse was allowed to explore the entire social test arena for a 10-min session. The amount of time spent in each chamber and the total traveled distance were recorded by the video-tracking system “Ethovision XT 8.5” (Noldus, Inc.). An entry was defined as all four paws in one chamber. Based on the amount of time spent in each chamber, a ‘sociability index’ (with a value of 0 meaning no preference) was calculated according to the following formula: Social index=((time_stranger / (time_stranger+time_empty)) * 100) – 50

**Tail suspension test (TST)**

The TST was performed following restraint stress as described ^26^. Briefly, animals were restrained for 2 h in a modified 50 ml, clear polypropylene tube (diameter 3 cm, 12 cm long) with multiple air holes for ventilation. 15 minutes after the restraint stress mice were tested in the tail suspension test. Behavioral despair in the TST was measured by suspending the mouse by the tail (attached to the bar by adhesive tape) on a horizontal aluminum bar attached to the top of a box-like enclosure (33×33×32 cm). The distance between the tip of the nose of the mouse and the floor was approximately 20 cm. Videos of mice were recorded and the time spent immobile during the 6 min was analyzed by the video tracking system “EthoVision XT 8.5” (Noldus, Inc.).

**Sucrose preference**

The sucrose preference test was performed as described earlier ^27^ using a two-bottle procedure, during which mice had free access to both water and a sucrose solution. Single housed animals were exposed in their regular home cage with the two standard water bottles: One with regular water, another with 2% sucrose solution. The first 2 days served as a habituation to sucrose solution. The results of next 24h were used for the evaluation of sucrose preference. Weight of each bottle was recorder before and after 24h period. Bottles were counterbalanced across the left and the right sides of the cage, and their position was alternated from test to test. Sucrose preference (%) was calculated as follows: preference = [sucrose solution intake (ml)/total fluid intake (ml)] × 100.

**Fear Conditioning**

Cognitive function was assessed in the contextual fear conditioning test implemented using a multi-conditioning system (TSE Systems GmbH, Bad Homburg, Germany). The test measures hippocampus-dependent contextual place memory. For training, an animal was placed inside a Plexiglas chamber (36×20×20 cm) with a removable shock grid made of stainless-steel rods (4 mm in diameter, spaced 6 mm apart). After 2 min, animals received a first electrical shock (0.4 mA, 2 s) and 15 s later a second shock with the same characteristics. Animal behavior was recorded by a video camera to monitor freezing behavior, defined as the lack of movement (excluding respiratory movements). The analysis of the freezing behavior was performed using video-tracking software (“EthovisionXT v.8.5”; Noldus Information Technology, Wageningen, The Netherlands). The contextual memory test was performed 24 h after training. Mice were monitored for freezing for 2 min in the same context chamber that was used for training. The cumulative duration (s) of freezing behavior during the 2 min of testing was used as a readout for fear memory.

**Chronic social defeat (CSD) stress**

In our study we have used CSD procedure, described earlier ^28^. In brief, every day for 10 days, mutant mice were introduced into a different home cage of an older, larger, and retired male breeder (thus aggressive) mouse of CD-1 strain. After period of fighting for a total of 30s, a mesh wall was introduced in the middle of the cage between the two mice allowing sensory but not physical contact for 24 h before repeating the same procedure with defeated mice encountering different CD-1 aggressors every time. The CD-1 aggressors were pre-trained to attack another mouse for 3 days before beginning CSD to standardize attack's latency. Age-matched mice from no-CSD group were housed in the same conditions (i.e. 2 per cage, separated by mesh partition) and were handled throughout 10 days by being individually placed 3 times a day in an empty cage for 30 sec. and then returned to home cage. Cages were maintained in environmentally controlled cabinets (Uniprotect NG by Zoonlab) but separate for each group.

**Social interaction test after CSD stress**

Social interaction test was performed 7 days after CSD as described elsewhere ^28^ with the following modifications: An Ethovision XT 8.5 (Noldus) video-tracking system was used to score social interaction with an unfamiliar social target. Testing arena was a white plastic open field (OF, 40 x 40 cm), maintained under low (30 lx) illumination. Each experimental mouse was introduced into the OF and its trajectory was tracked for two consecutive sessions of 2.5 min. During the first session (“no target”) the open field contained an empty wire mesh cage (10 x 6.5 cm) located at the middle of one of OF walls, touching it. During the second session (“target”), the conditions were identical except that a social target animal (an unfamiliar CD1 male mouse) had been introduced into the cage. Between the 2 sessions, the experimental mouse was removed from the arena, and was placed back into its home cage for approximately one min. The video-tracking data from both the “no target” and “target” conditions were used to measure the time spent by the experimental mouse in the “interaction zone” (an 8 cm-wide corridor surrounding the cage). A social interaction index (SI) was calculated as 100 × (time in interaction zone, CD-1 mouse present)/(time in interaction zone, empty cage). Finally, based on their SI mice were classified into Resilient (SI index ≥ 100) or Susceptible (SI index < 100) (Golden et al., 2011).

**References**

1. Sheehan, D.V., Lecrubier, Y., Sheehan, K.H., Amorim, P., Janavs, J., Weiller, E., Hergueta, T., Baker, R., and Dunbar, G.C. (1998). The Mini-International Neuropsychiatric Interview (M.I.N.I.): the development and validation of a structured diagnostic psychiatric interview for DSM-IV and ICD-10. The Journal of clinical psychiatry *59 Suppl 20*, 22-33;quiz 34-57.

2. Kumari, V., and Gray, J.A. (1999). Smoking withdrawal, nicotine dependence and prepulse inhibition of the acoustic startle reflex. Psychopharmacology *141*, 11-15. 10.1007/s002130050800.

3. Boxhoorn, S., Lopez, E., Schmidt, C., Schulze, D., Hanig, S., and Freitag, C.M. (2018). Attention profiles in autism spectrum disorder and subtypes of attention-deficit/hyperactivity disorder. Eur Child Adolesc Psychiatry *27*, 1433-1447. 10.1007/s00787-018-1138-8.

4. Leclercq, M., Zimmermann, P.,. (2002). Applied Neuropsychology of Attention. Theory, Diagnosis and Rehabilitation. (Psychology Press).

5. American Electroencephalographic Society guidelines for standard electrode position nomenclature. (1991). J Clin Neurophysiol *8*, 200-202.

6. Klem, G.H., Luders, H.O., Jasper, H.H., and Elger, C. (1999). The ten-twenty electrode system of the International Federation. The International Federation of Clinical Neurophysiology. Electroencephalogr Clin Neurophysiol Suppl *52*, 3-6.

7. Berardelli, A., Abbruzzese, G., Chen, R., Orth, M., Ridding, M.C., Stinear, C., Suppa, A., Trompetto, C., and Thompson, P.D. (2008). Consensus paper on short-interval intracortical inhibition and other transcranial magnetic stimulation intracortical paradigms in movement disorders. Brain stimulation *1*, 183-191. 10.1016/j.brs.2008.06.005.

8. Chirumamilla, V.C., Gonzalez-Escamilla, G., Koirala, N., Bonertz, T., von Grotthus, S., Muthuraman, M., and Groppa, S. (2019). Cortical Excitability Dynamics During Fear Processing. Frontiers in neuroscience *13*, 568. 10.3389/fnins.2019.00568.

9. van der Veen, R.C., Hinton, D.R., Incardonna, F., and Hofman, F.M. (1997). Extensive peroxynitrite activity during progressive stages of central nervous system inflammation. Journal of neuroimmunology *77*, 1-7. 10.1016/s0165-5728(97)00013-1.

10. Muthuraman, M., Raethjen, J., Koirala, N., Anwar, A.R., Mideksa, K.G., Elble, R., Groppa, S., and Deuschl, G. (2018). Cerebello-cortical network fingerprints differ between essential, Parkinson's and mimicked tremors. Brain : a journal of neurology *141*, 1770-1781. 10.1093/brain/awy098.

11. Van Veen, B.D., van Drongelen, W., Yuchtman, M., and Suzuki, A. (1997). Localization of brain electrical activity via linearly constrained minimum variance spatial filtering. IEEE Trans Biomed Eng *44*, 867-880. 10.1109/10.623056.

12. Desikan, R.S., Segonne, F., Fischl, B., Quinn, B.T., Dickerson, B.C., Blacker, D., Buckner, R.L., Dale, A.M., Maguire, R.P., Hyman, B.T., et al. (2006). An automated labeling system for subdividing the human cerebral cortex on MRI scans into gyral based regions of interest. NeuroImage *31*, 968-980. 10.1016/j.neuroimage.2006.01.021.

13. Anwar, A.R., Muthalib, M., Perrey, S., Galka, A., Granert, O., Wolff, S., Heute, U., Deuschl, G., Raethjen, J., and Muthuraman, M. (2016). Effective Connectivity of Cortical Sensorimotor Networks During Finger Movement Tasks: A Simultaneous fNIRS, fMRI, EEG Study. Brain Topogr *29*, 645-660. 10.1007/s10548-016-0507-1.

14. Whitfield-Gabrieli, S., and Nieto-Castanon, A. (2012). Conn: a functional connectivity toolbox for correlated and anticorrelated brain networks. Brain Connect *2*, 125-141. 10.1089/brain.2012.0073.

15. Girvan, M., and Newman, M.E. (2002). Community structure in social and biological networks. Proceedings of the National Academy of Sciences of the United States of America *99*, 7821-7826. 10.1073/pnas.122653799.

16. Newman, M.E. (2006). Modularity and community structure in networks. Proceedings of the National Academy of Sciences of the United States of America *103*, 8577-8582. 10.1073/pnas.0601602103.

17. Erk, S., Meyer-Lindenberg, A., Schnell, K., Opitz von Boberfeld, C., Esslinger, C., Kirsch, P., Grimm, O., Arnold, C., Haddad, L., Witt, S.H., et al. (2010). Brain function in carriers of a genome-wide supported bipolar disorder variant. Archives of general psychiatry *67*, 803-811. 10.1001/archgenpsychiatry.2010.94.

18. Erk, S., Meyer-Lindenberg, A., Linden, D.E.J., Lancaster, T., Mohnke, S., Grimm, O., Degenhardt, F., Holmans, P., Pocklington, A., Schmierer, P., et al. (2014). Replication of brain function effects of a genome-wide supported psychiatric risk variant in the CACNA1C gene and new multi-locus effects. NeuroImage *94*, 147-154. 10.1016/j.neuroimage.2014.03.007.

19. de Quervain, D.J., and Papassotiropoulos, A. (2006). Identification of a genetic cluster influencing memory performance and hippocampal activity in humans. Proceedings of the National Academy of Sciences of the United States of America *103*, 4270-4274. 10.1073/pnas.0510212103.

20. Schaefer, A., Kong, R., Gordon, E.M., Laumann, T.O., Zuo, X.N., Holmes, A.J., Eickhoff, S.B., and Yeo, B.T.T. (2018). Local-Global Parcellation of the Human Cerebral Cortex from Intrinsic Functional Connectivity MRI. Cerebral cortex *28*, 3095-3114. 10.1093/cercor/bhx179.

21. Chirumamilla, V.C., Gonzalez-Escamilla, G., Meyer, B., Anwar, A.R., Ding, H., Radetz, A., Bonertz, T., Groppa, S., and Muthuraman, M. (2022). Inhibitory and excitatory responses in the dorso-medial prefrontal cortex during threat processing. Frontiers in neuroscience *16*, 1065469. 10.3389/fnins.2022.1065469.

22. Thalman, C., Horta, G., Qiao, L., Endle, H., Tegeder, I., Cheng, H., Laube, G., Sigrudsson, T., Hauser, M.J., Tenzer, S., et al. (2018). Synaptic phospholipids as a new target for cortical hyperexcitability and E/I balance in psychiatric disorders. Molecular psychiatry *23*, 1699-1710. 10.1038/s41380-018-0053-1.

23. Trimbuch, T., Beed, P., Vogt, J., Schuchmann, S., Maier, N., Kintscher, M., Breustedt, J., Schuelke, M., Streu, N., Kieselmann, O., et al. (2009). Synaptic PRG-1 modulates excitatory transmission via lipid phosphate-mediated signaling. Cell *138*, 1222-1235.

24. O'Neill, P.K., Gordon, J.A., and Sigurdsson, T. (2013). Theta oscillations in the medial prefrontal cortex are modulated by spatial working memory and synchronize with the hippocampus through its ventral subregion. The Journal of neuroscience : the official journal of the Society for Neuroscience *33*, 14211-14224. 10.1523/JNEUROSCI.2378-13.2013.

25. Radyushkin, K., El-Kordi, A., Boretius, S., Castaneda, S., Ronnenberg, A., Reim, K., Bickeboller, H., Frahm, J., Brose, N., and Ehrenreich, H. (2010). Complexin2 null mutation requires a 'second hit' for induction of phenotypic changes relevant to schizophrenia. Genes, brain, and behavior *9*, 592-602. 10.1111/j.1601-183X.2010.00590.x.

26. Vogt, J., Yang, J.W., Mobascher, A., Cheng, J., Li, Y., Liu, X., Baumgart, J., Thalman, C., Kirischuk, S., Unichenko, P., et al. (2016). Molecular cause and functional impact of altered synaptic lipid signaling due to a prg-1 gene SNP. EMBO molecular medicine *8*, 25-38. 10.15252/emmm.201505677.

27. Jamain, S., Radyushkin, K., Hammerschmidt, K., Granon, S., Boretius, S., Varoqueaux, F., Ramanantsoa, N., Gallego, J., Ronnenberg, A., Winter, D., et al. (2008). Reduced social interaction and ultrasonic communication in a mouse model of monogenic heritable autism. Proceedings of the National Academy of Sciences of the United States of America *105*, 1710-1715. 10.1073/pnas.0711555105.

28. Golden, S.A., Covington, H.E., 3rd, Berton, O., and Russo, S.J. (2011). A standardized protocol for repeated social defeat stress in mice. Nature protocols *6*, 1183-1191. 10.1038/nprot.2011.361.
