# Supplementary material for: Altered cortical synaptic lipid signaling leads to intermediate phenotypes of mental disorders
Source: Mol Psychiatry. 2024 May 28;29(11):3537–52. doi: 10.1038/s41380-024-02598-2 (PMC11541086; doi:10.1038/s41380-024-02598-2)
Supplement: Supplementary file 2 — Supplementary Figures 1–3 [file 41380_2024_2598_MOESM2_ESM.pptx]

## Slide 1
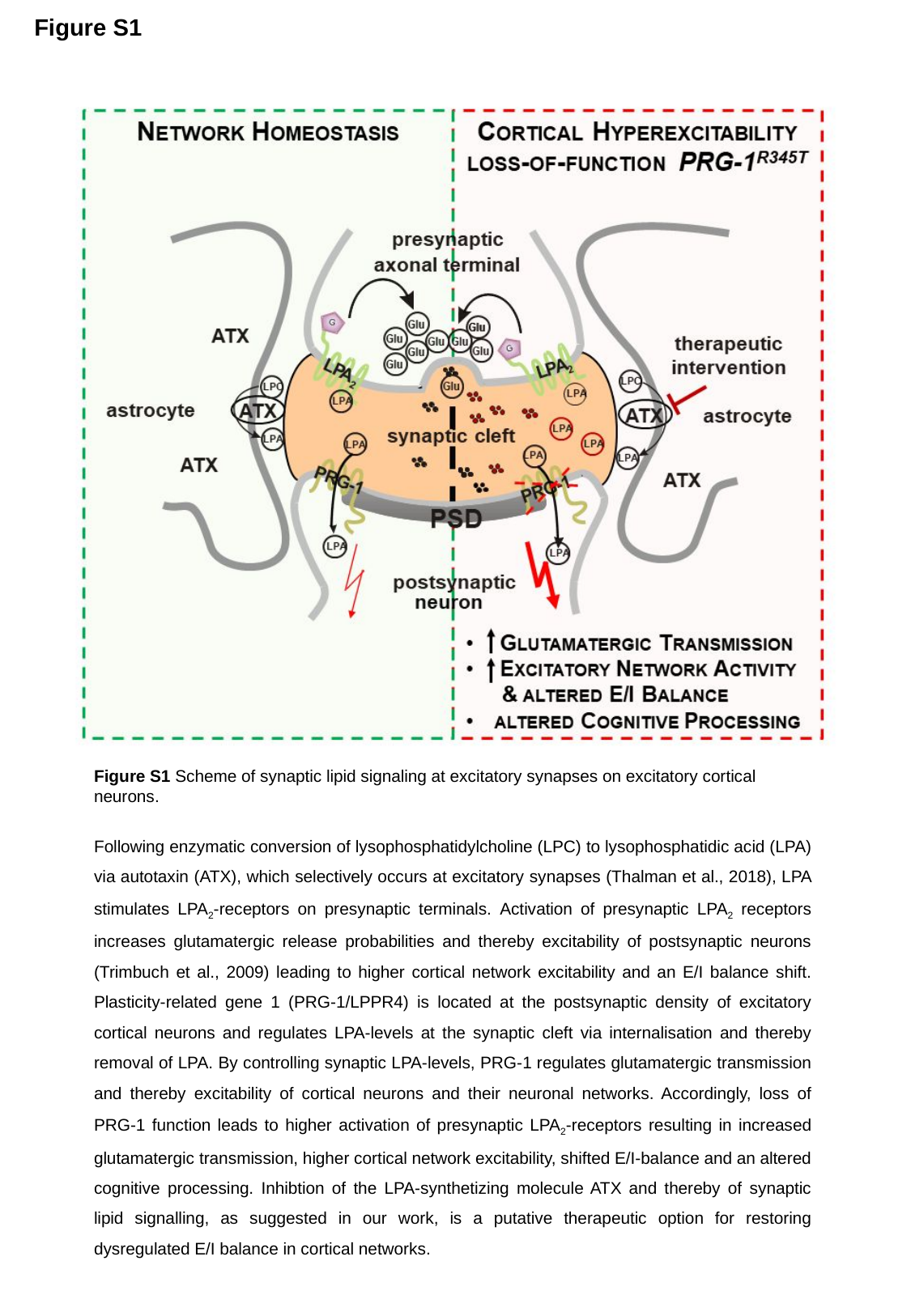

Figure S1
Figure S1 Scheme of synaptic lipid signaling at excitatory synapses on excitatory cortical neurons.
Following enzymatic conversion of lysophosphatidylcholine (LPC) to lysophosphatidic acid (LPA) via autotaxin (ATX), which selectively occurs at excitatory synapses (Thalman et al., 2018), LPA stimulates LPA2-receptors on presynaptic terminals. Activation of presynaptic LPA2 receptors increases glutamatergic release probabilities and thereby excitability of postsynaptic neurons (Trimbuch et al., 2009) leading to higher cortical network excitability and an E/I balance shift. Plasticity-related gene 1 (PRG-1/LPPR4) is located at the postsynaptic density of excitatory cortical neurons and regulates LPA-levels at the synaptic cleft via internalisation and thereby removal of LPA. By controlling synaptic LPA-levels, PRG-1 regulates glutamatergic transmission and thereby excitability of cortical neurons and their neuronal networks. Accordingly, loss of PRG-1 function leads to higher activation of presynaptic LPA2-receptors resulting in increased glutamatergic transmission, higher cortical network excitability, shifted E/I-balance and an altered cognitive processing. Inhibtion of the LPA-synthetizing molecule ATX and thereby of synaptic lipid signalling, as suggested in our work, is a putative therapeutic option for restoring dysregulated E/I balance in cortical networks.

## Slide 2
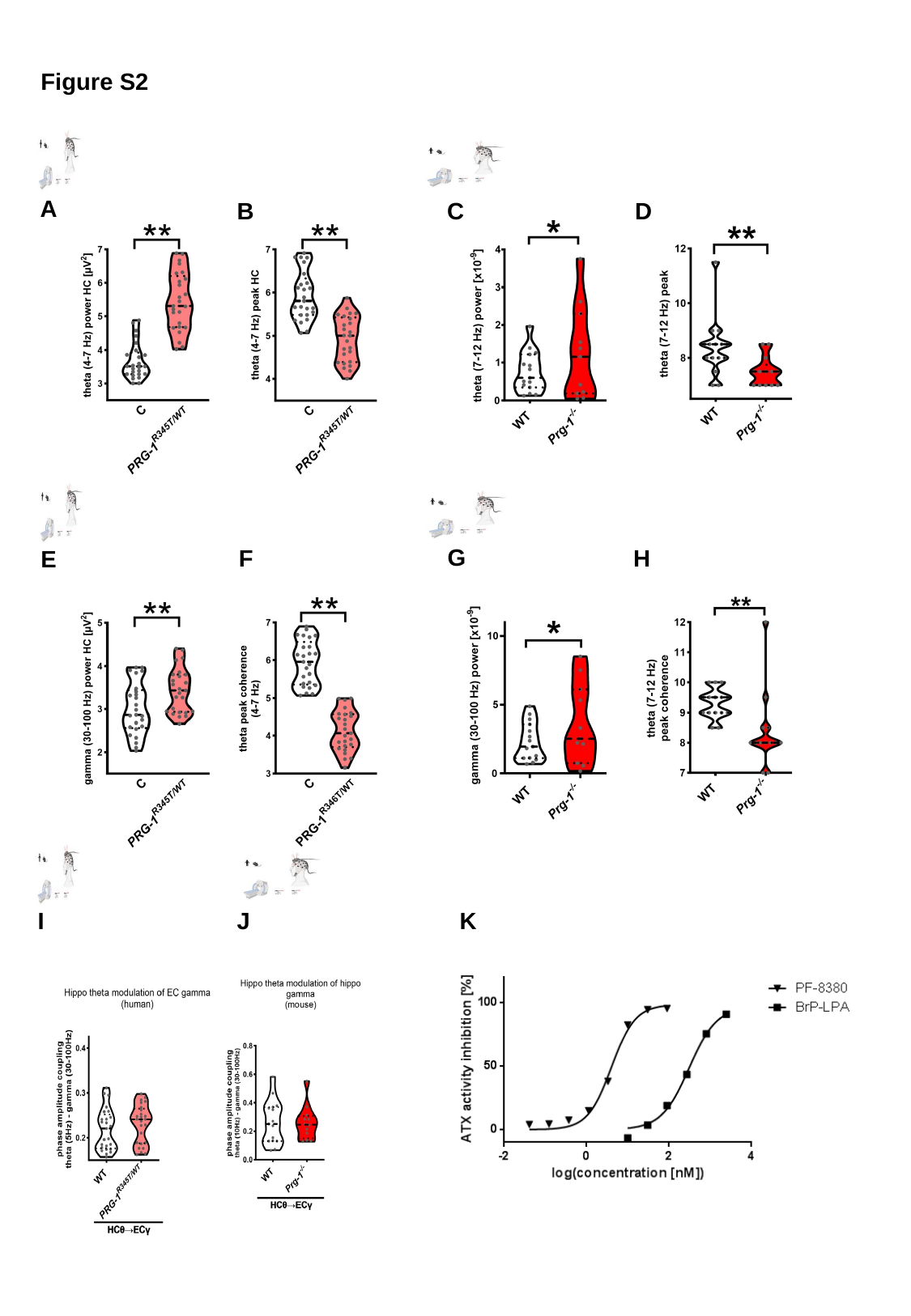

Figure S2
A
B
C
D
G
H
F
E
K
J
I

## Slide 3
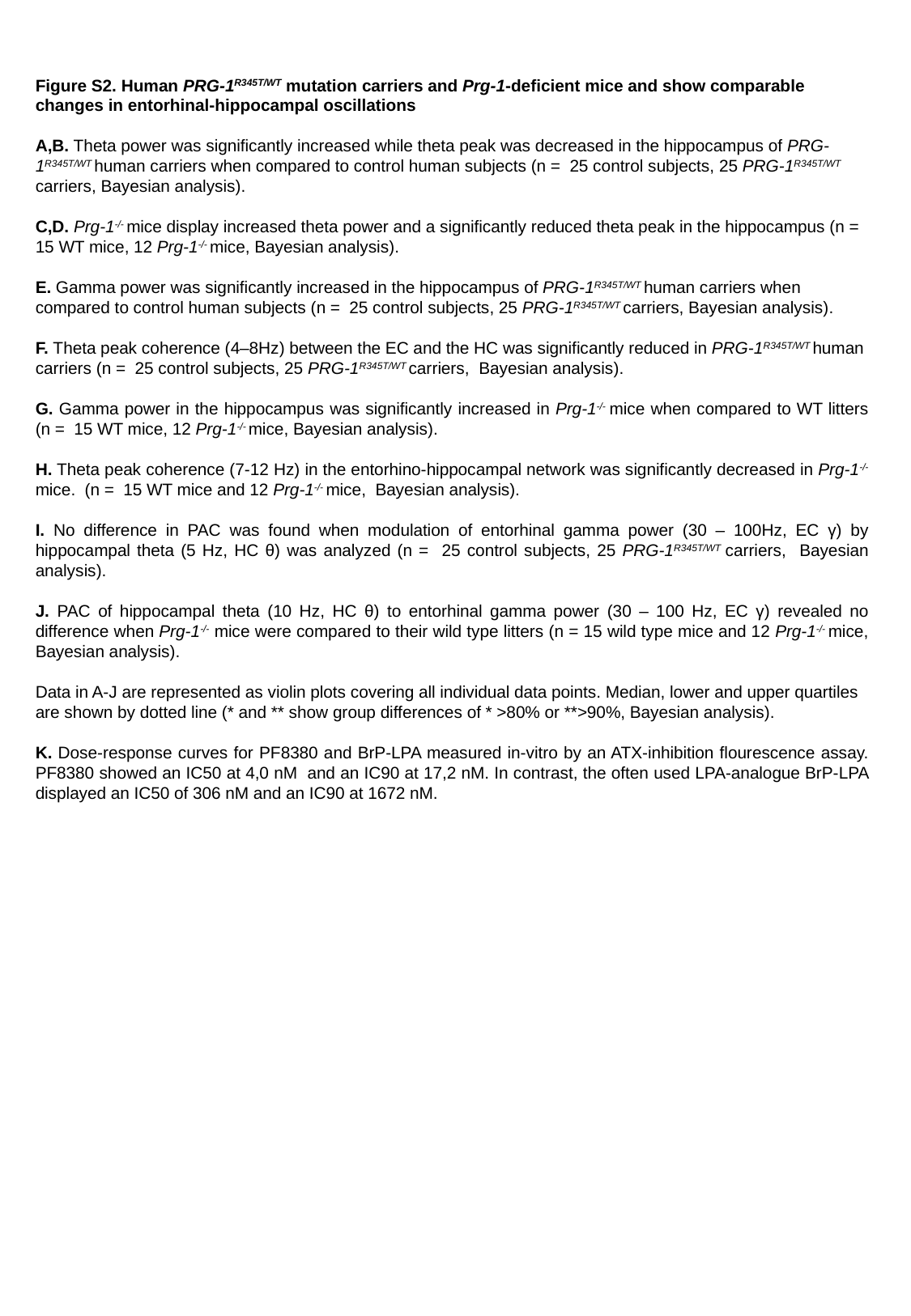

Figure S2. Human PRG-1R345T/WT mutation carriers and Prg-1-deficient mice and show comparable changes in entorhinal-hippocampal oscillations
A,B. Theta power was significantly increased while theta peak was decreased in the hippocampus of PRG-1R345T/WT human carriers when compared to control human subjects (n = 25 control subjects, 25 PRG-1R345T/WT carriers, Bayesian analysis).
C,D. Prg-1-/- mice display increased theta power and a significantly reduced theta peak in the hippocampus (n = 15 WT mice, 12 Prg-1-/- mice, Bayesian analysis).
E. Gamma power was significantly increased in the hippocampus of PRG-1R345T/WT human carriers when compared to control human subjects (n = 25 control subjects, 25 PRG-1R345T/WT carriers, Bayesian analysis).
F. Theta peak coherence (4–8Hz) between the EC and the HC was significantly reduced in PRG-1R345T/WT human carriers (n = 25 control subjects, 25 PRG-1R345T/WT carriers, Bayesian analysis).
G. Gamma power in the hippocampus was significantly increased in Prg-1-/- mice when compared to WT litters (n = 15 WT mice, 12 Prg-1-/- mice, Bayesian analysis).
H. Theta peak coherence (7-12 Hz) in the entorhino-hippocampal network was significantly decreased in Prg-1-/- mice. (n = 15 WT mice and 12 Prg-1-/- mice, Bayesian analysis).
I. No difference in PAC was found when modulation of entorhinal gamma power (30 – 100Hz, EC γ) by hippocampal theta (5 Hz, HC θ) was analyzed (n = 25 control subjects, 25 PRG-1R345T/WT carriers, Bayesian analysis).
J. PAC of hippocampal theta (10 Hz, HC θ) to entorhinal gamma power (30 – 100 Hz, EC γ) revealed no difference when Prg-1-/- mice were compared to their wild type litters (n = 15 wild type mice and 12 Prg-1-/- mice, Bayesian analysis).
Data in A-J are represented as violin plots covering all individual data points. Median, lower and upper quartiles are shown by dotted line (* and ** show group differences of * >80% or **>90%, Bayesian analysis).
K. Dose-response curves for PF8380 and BrP-LPA measured in-vitro by an ATX-inhibition flourescence assay. PF8380 showed an IC50 at 4,0 nM and an IC90 at 17,2 nM. In contrast, the often used LPA-analogue BrP-LPA displayed an IC50 of 306 nM and an IC90 at 1672 nM.

## Slide 4
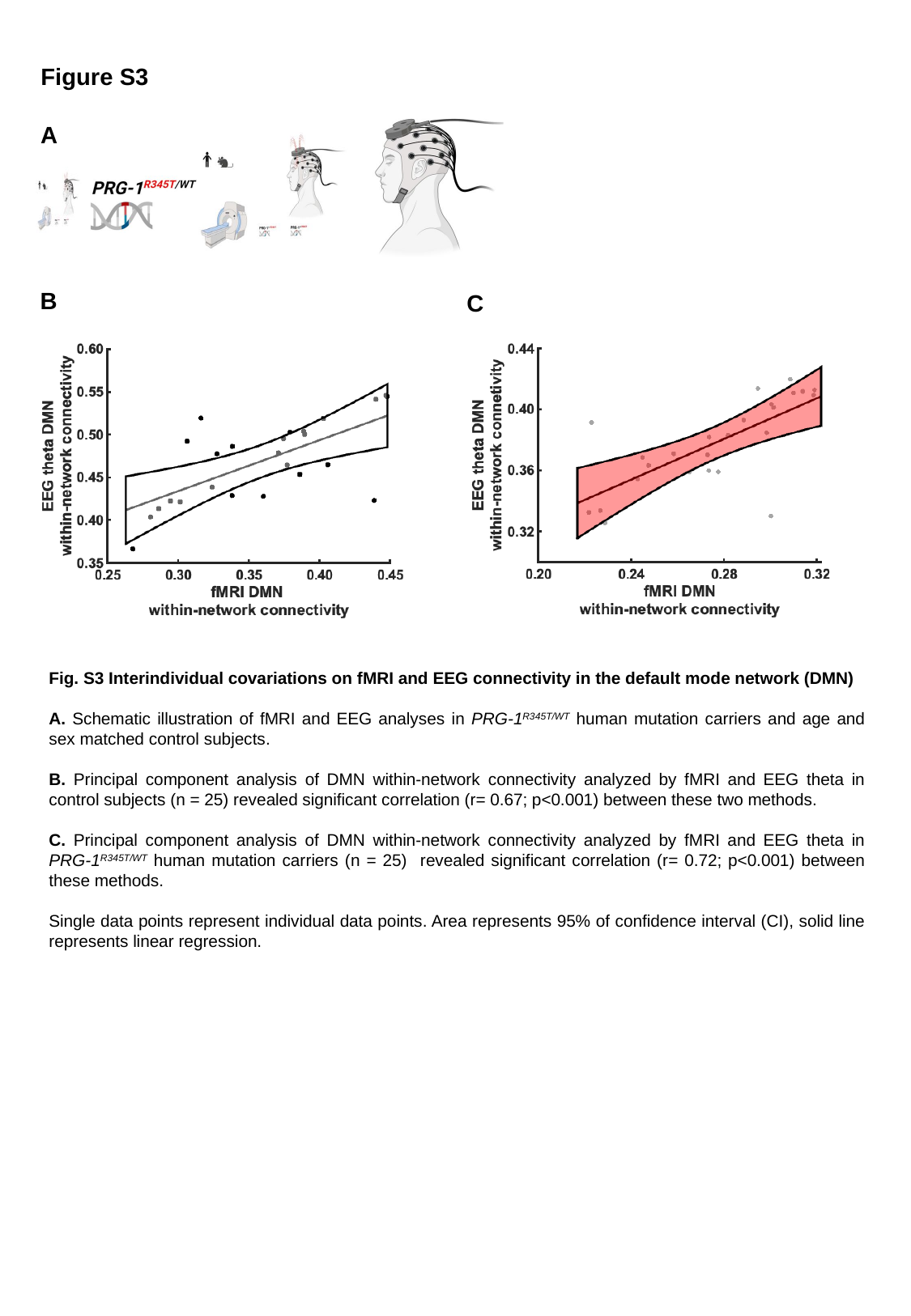

Figure S3
A
B
C
Fig. S3 Interindividual covariations on fMRI and EEG connectivity in the default mode network (DMN)
A. Schematic illustration of fMRI and EEG analyses in PRG-1R345T/WT human mutation carriers and age and sex matched control subjects.
B. Principal component analysis of DMN within-network connectivity analyzed by fMRI and EEG theta in control subjects (n = 25) revealed significant correlation (r= 0.67; p<0.001) between these two methods.
C. Principal component analysis of DMN within-network connectivity analyzed by fMRI and EEG theta in PRG-1R345T/WT human mutation carriers (n = 25) revealed significant correlation (r= 0.72; p<0.001) between these methods.
Single data points represent individual data points. Area represents 95% of confidence interval (CI), solid line represents linear regression.

## Slide 5
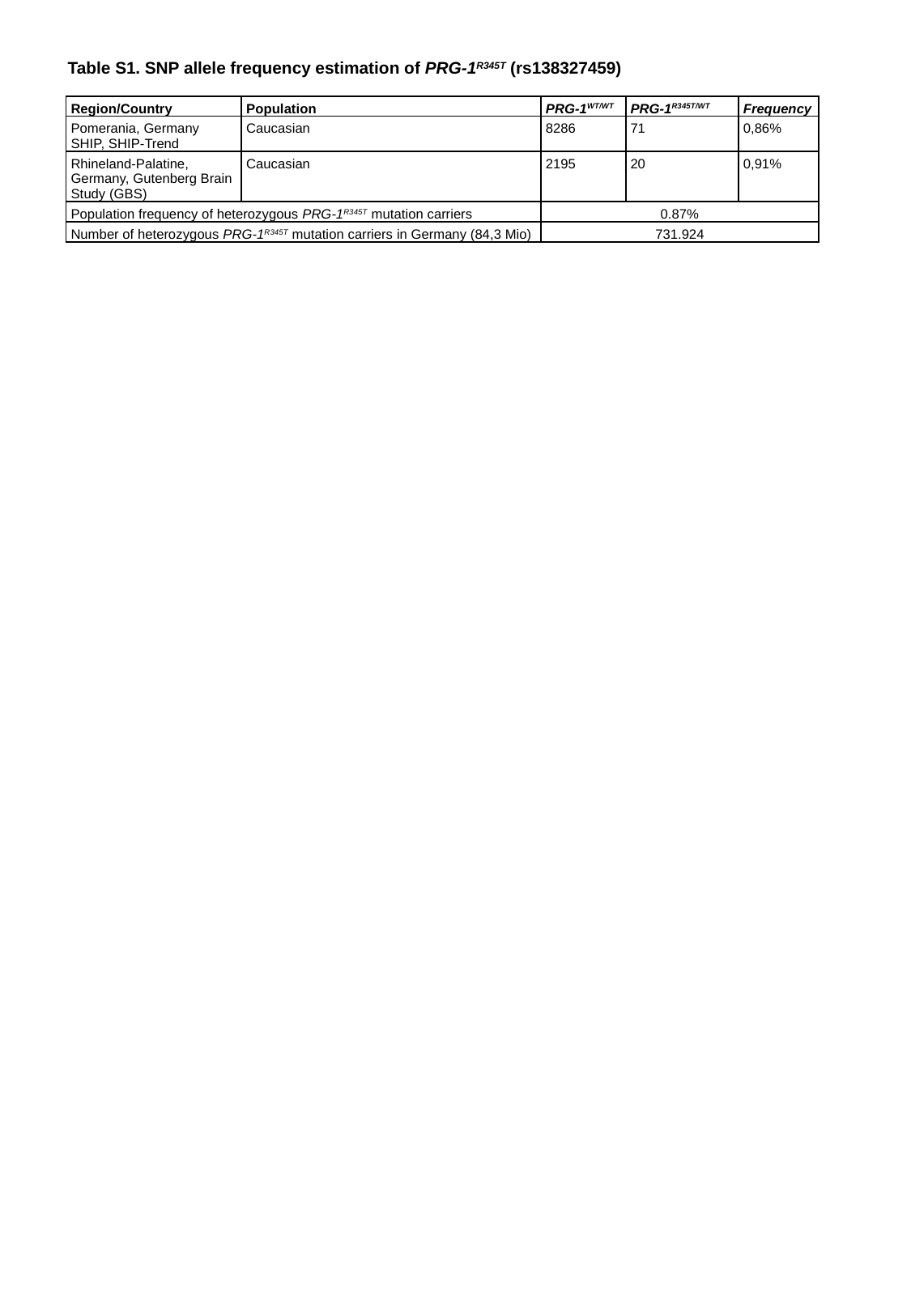

Table S1. SNP allele frequency estimation of PRG-1R345T (rs138327459)
| Region/Country | Population | PRG-1WT/WT | PRG-1R345T/WT | Frequency |
| --- | --- | --- | --- | --- |
| Pomerania, Germany SHIP, SHIP-Trend | Caucasian | 8286 | 71 | 0,86% |
| Rhineland-Palatine, Germany, Gutenberg Brain Study (GBS) | Caucasian | 2195 | 20 | 0,91% |
| Population frequency of heterozygous PRG-1R345T mutation carriers | | 0.87% | | |
| Number of heterozygous PRG-1R345T mutation carriers in Germany (84,3 Mio) | | 731.924 | | |

## Slide 6
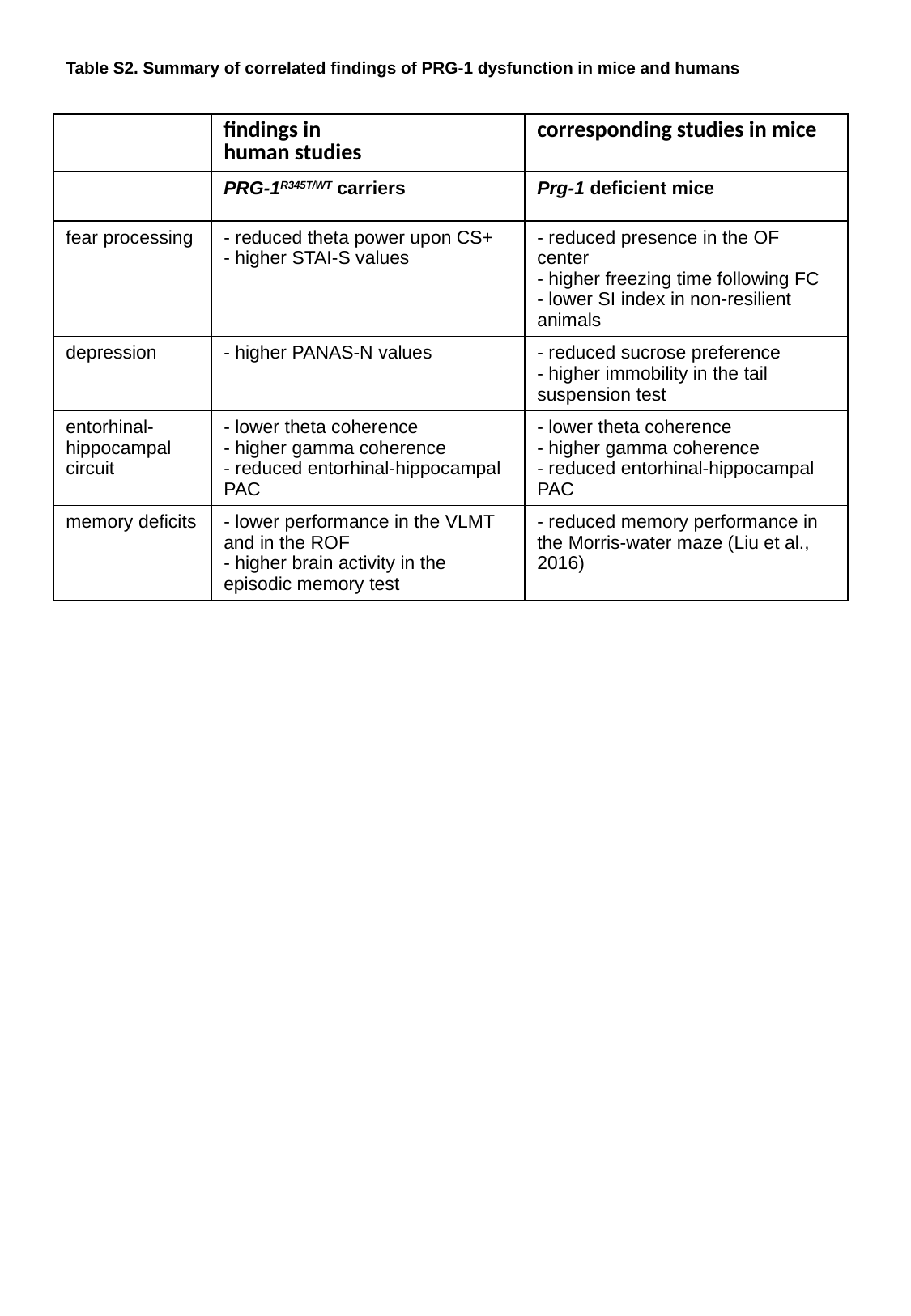

Table S2. Summary of correlated findings of PRG-1 dysfunction in mice and humans
| | findings in human studies | corresponding studies in mice |
| --- | --- | --- |
| | PRG-1R345T/WT carriers | Prg-1 deficient mice |
| fear processing | - reduced theta power upon CS+ - higher STAI-S values | - reduced presence in the OF center - higher freezing time following FC - lower SI index in non-resilient animals |
| depression | - higher PANAS-N values | - reduced sucrose preference - higher immobility in the tail suspension test |
| entorhinal-hippocampal circuit | - lower theta coherence - higher gamma coherence - reduced entorhinal-hippocampal PAC | - lower theta coherence - higher gamma coherence - reduced entorhinal-hippocampal PAC |
| memory deficits | - lower performance in the VLMT and in the ROF - higher brain activity in the episodic memory test | - reduced memory performance in the Morris-water maze (Liu et al., 2016) |

## Slide 7
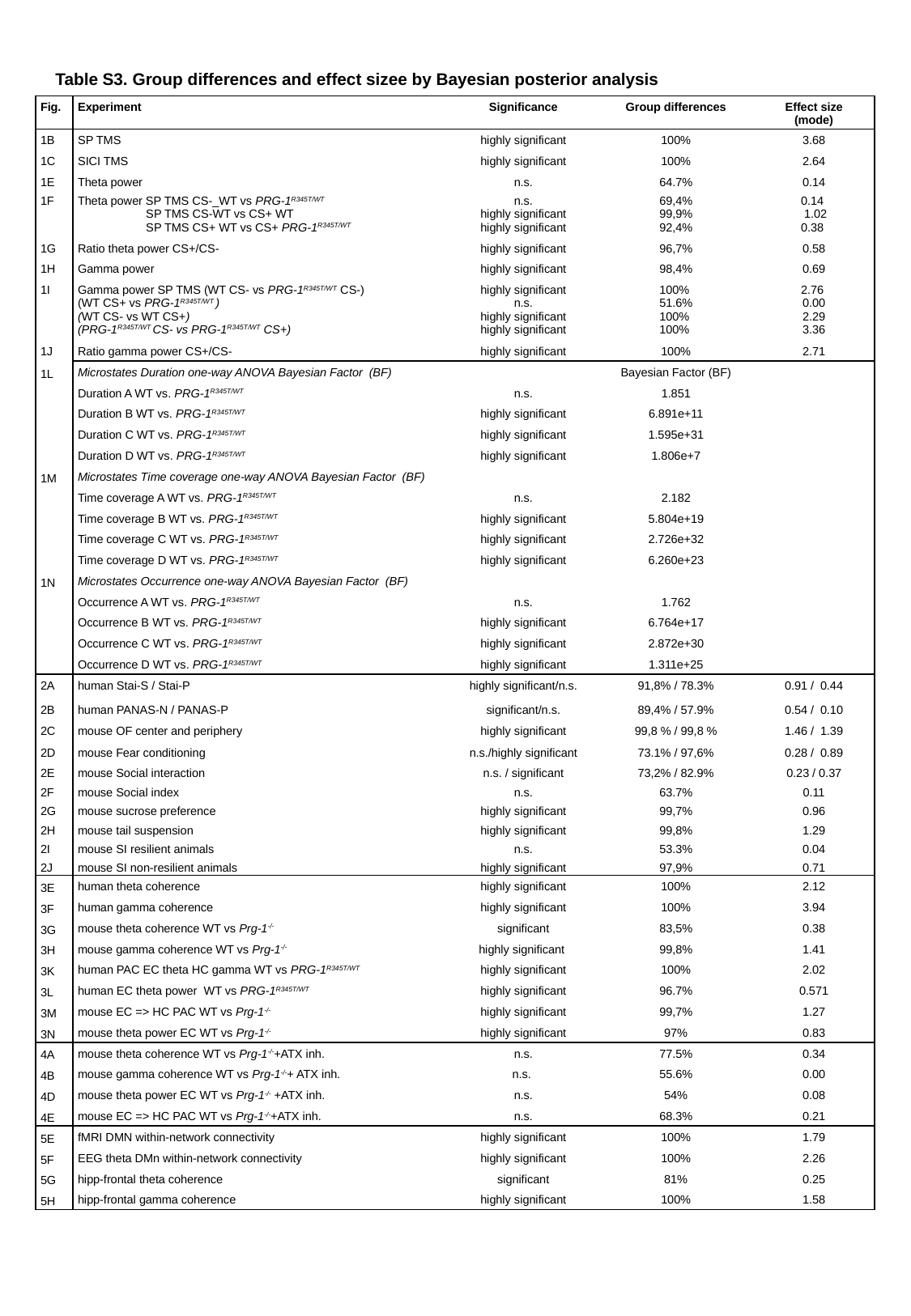

Table S3. Group differences and effect sizee by Bayesian posterior analysis
| Fig. | Experiment | Significance | Group differences | Effect size (mode) |
| --- | --- | --- | --- | --- |
| 1B | SP TMS | highly significant | 100% | 3.68 |
| 1C | SICI TMS | highly significant | 100% | 2.64 |
| 1E | Theta power | n.s. | 64.7% | 0.14 |
| 1F | Theta power SP TMS CS-\_WT vs PRG-1R345T/WT SP TMS CS-WT vs CS+ WT SP TMS CS+ WT vs CS+ PRG-1R345T/WT | n.s. highly significant highly significant | 69,4% 99,9% 92,4% | 0.14 1.02 0.38 |
| 1G | Ratio theta power CS+/CS- | highly significant | 96,7% | 0.58 |
| 1H | Gamma power | highly significant | 98,4% | 0.69 |
| 1I | Gamma power SP TMS (WT CS- vs PRG-1R345T/WT CS-) (WT CS+ vs PRG-1R345T/WT ) (WT CS- vs WT CS+) (PRG-1R345T/WT CS- vs PRG-1R345T/WT CS+) | highly significant n.s. highly significant highly significant | 100% 51.6% 100% 100% | 2.76 0.00 2.29 3.36 |
| 1J | Ratio gamma power CS+/CS- | highly significant | 100% | 2.71 |
| 1L | Microstates Duration one-way ANOVA Bayesian Factor (BF) | | Bayesian Factor (BF) | |
| | Duration A WT vs. PRG-1R345T/WT | n.s. | 1.851 | |
| | Duration B WT vs. PRG-1R345T/WT | highly significant | 6.891e+11 | |
| | Duration C WT vs. PRG-1R345T/WT | highly significant | 1.595e+31 | |
| | Duration D WT vs. PRG-1R345T/WT | highly significant | 1.806e+7 | |
| 1M | Microstates Time coverage one-way ANOVA Bayesian Factor (BF) | | | |
| | Time coverage A WT vs. PRG-1R345T/WT | n.s. | 2.182 | |
| | Time coverage B WT vs. PRG-1R345T/WT | highly significant | 5.804e+19 | |
| | Time coverage C WT vs. PRG-1R345T/WT | highly significant | 2.726e+32 | |
| | Time coverage D WT vs. PRG-1R345T/WT | highly significant | 6.260e+23 | |
| 1N | Microstates Occurrence one-way ANOVA Bayesian Factor (BF) | | | |
| | Occurrence A WT vs. PRG-1R345T/WT | n.s. | 1.762 | |
| | Occurrence B WT vs. PRG-1R345T/WT | highly significant | 6.764e+17 | |
| | Occurrence C WT vs. PRG-1R345T/WT | highly significant | 2.872e+30 | |
| | Occurrence D WT vs. PRG-1R345T/WT | highly significant | 1.311e+25 | |
| 2A | human Stai-S / Stai-P | highly significant/n.s. | 91,8% / 78.3% | 0.91 / 0.44 |
| 2B | human PANAS-N / PANAS-P | significant/n.s. | 89,4% / 57.9% | 0.54 / 0.10 |
| 2C | mouse OF center and periphery | highly significant | 99,8 % / 99,8 % | 1.46 / 1.39 |
| 2D | mouse Fear conditioning | n.s./highly significant | 73.1% / 97,6% | 0.28 / 0.89 |
| 2E | mouse Social interaction | n.s. / significant | 73,2% / 82.9% | 0.23 / 0.37 |
| 2F | mouse Social index | n.s. | 63.7% | 0.11 |
| 2G | mouse sucrose preference | highly significant | 99,7% | 0.96 |
| 2H | mouse tail suspension | highly significant | 99,8% | 1.29 |
| 2I | mouse SI resilient animals | n.s. | 53.3% | 0.04 |
| 2J | mouse SI non-resilient animals | highly significant | 97,9% | 0.71 |
| 3E | human theta coherence | highly significant | 100% | 2.12 |
| 3F | human gamma coherence | highly significant | 100% | 3.94 |
| 3G | mouse theta coherence WT vs Prg-1-/- | significant | 83,5% | 0.38 |
| 3H | mouse gamma coherence WT vs Prg-1-/- | highly significant | 99,8% | 1.41 |
| 3K | human PAC EC theta HC gamma WT vs PRG-1R345T/WT | highly significant | 100% | 2.02 |
| 3L | human EC theta power WT vs PRG-1R345T/WT | highly significant | 96.7% | 0.571 |
| 3M | mouse EC => HC PAC WT vs Prg-1-/- | highly significant | 99,7% | 1.27 |
| 3N | mouse theta power EC WT vs Prg-1-/- | highly significant | 97% | 0.83 |
| 4A | mouse theta coherence WT vs Prg-1-/-+ATX inh. | n.s. | 77.5% | 0.34 |
| 4B | mouse gamma coherence WT vs Prg-1-/-+ ATX inh. | n.s. | 55.6% | 0.00 |
| 4D | mouse theta power EC WT vs Prg-1-/- +ATX inh. | n.s. | 54% | 0.08 |
| 4E | mouse EC => HC PAC WT vs Prg-1-/-+ATX inh. | n.s. | 68.3% | 0.21 |
| 5E | fMRI DMN within-network connectivity | highly significant | 100% | 1.79 |
| 5F | EEG theta DMn within-network connectivity | highly significant | 100% | 2.26 |
| 5G | hipp-frontal theta coherence | significant | 81% | 0.25 |
| 5H | hipp-frontal gamma coherence | highly significant | 100% | 1.58 |

## Slide 8
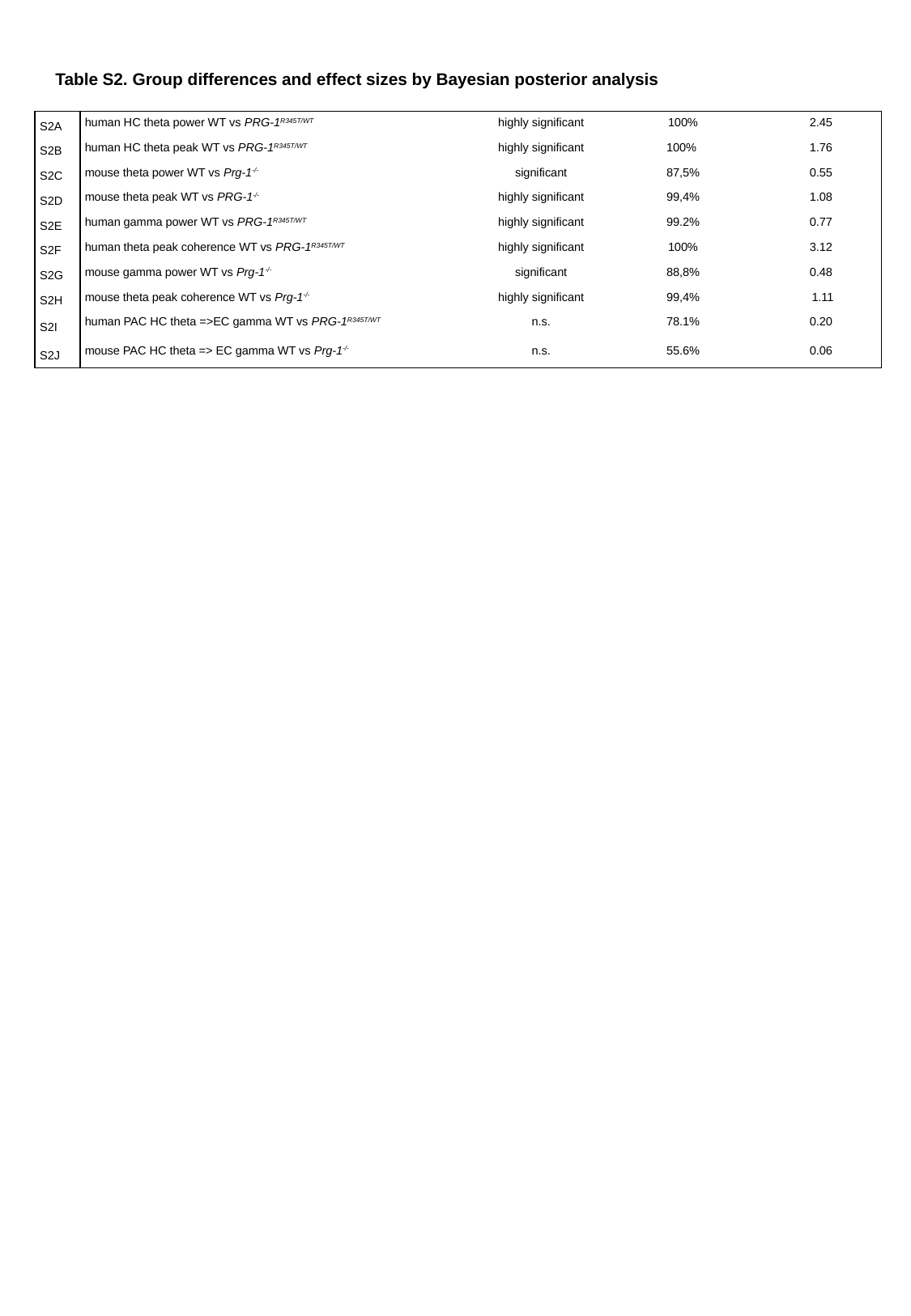

Table S2. Group differences and effect sizes by Bayesian posterior analysis
| S2A | human HC theta power WT vs PRG-1R345T/WT/WT/WT | highly significant | 100% | 2.45 |
| --- | --- | --- | --- | --- |
| S2B | human HC theta peak WT vs PRG-1R345T/WT | highly significant | 100% | 1.76 |
| S2C | mouse theta power WT vs Prg-1-/- | significant | 87,5% | 0.55 |
| S2D | mouse theta peak WT vs PRG-1-/- | highly significant | 99,4% | 1.08 |
| S2E | human gamma power WT vs PRG-1R345T/WT | highly significant | 99.2% | 0.77 |
| S2F | human theta peak coherence WT vs PRG-1R345T/WT | highly significant | 100% | 3.12 |
| S2G | mouse gamma power WT vs Prg-1-/- | significant | 88,8% | 0.48 |
| S2H | mouse theta peak coherence WT vs Prg-1-/- | highly significant | 99,4% | 1.11 |
| S2I | human PAC HC theta =>EC gamma WT vs PRG-1R345T/WT | n.s. | 78.1% | 0.20 |
| S2J | mouse PAC HC theta => EC gamma WT vs Prg-1-/- | n.s. | 55.6% | 0.06 |
